# Interaction of blood-entry components, network pharmacology and transcriptomics to elucidate the mechanism of Wentong plaster in treating primary dysmenorrhea

**DOI:** 10.3389/fphar.2025.1591558

**Published:** 2025-07-02

**Authors:** Zongtong Yang, Ziqi Jiao, Cheng Wang, Xiaojing Li, Mengyu Yuan, Zaiyun Sui, Wenhui Wang, Wenjing Hou

**Affiliations:** ^1^ Institute of Pharmacology of Traditional Chinese Medicine, Shandong Academy of Chinese Medicine, Jinan, China; ^2^ College of Pharmacy, Jining Medical University, Jining, China; ^3^ College of Veterinary Medicine, Gansu Agricultural University, Lanzhou, China

**Keywords:** Wentong plaster, primary dysmenorrhea, mechanism, network pharmacology, transcriptomics

## Abstract

**Introduction:**

Primary dysmenorrhea (PD) is characterized by pain during the menstrual cycle, affects women's health. Our group developed a traditional Chinese medicine plaster (Wentong plaster, WTT) for the treatment of PD. However, the underlying mechanisms have not yet been elucidated.

**Methods:**

In this study, the blood-entry components of WTT were detected using UPLC-Q-Exactive Orbitrap-MS, and the therapeutic functions of WTT on PD were evaluated by the writhing response, pathological analysis, and the levels of estrogen, nitric oxide, progesterone, among other indicators. Network pharmacology and transcriptomics were used to elucidate the underlying mechanisms. Finally, enzyme-linked immunosorbent assay and western blotting were used to determine the levels of relevant indicators.

**Results:**

Our findings indicate that 49 original blood-entry components were detected. Meanwhile, WTT upregulated the level of NO, and downregulated the levels of PGF2α, PGE2, estrogen, and progesterone, thereby increasing blood flow, alleviating inflammatory responses, and inhibiting the writhing response. Results from network pharmacology and transcriptomics analyses indicated that WTT could increase the expression of Lcn2 and decrease the expression of Cxcl6 and IL-17, thereby regulating the IL-17 signaling pathway, and alleviating inflammation to treat PD.

**Conclusion:**

WTT mainly down-regulates the levels of Cxcl6 and IL-17 and up-regulates the expression of Lcn2, further regulates the IL-17 signaling pathway to alleviate inflammation, ultimately treating PD. This study provides a basis for further research on the mechanism of WTT, and offers a reference for its clinical application.

## 1 Introduction

Dysmenorrhea, is a common gynecological condition classified as either primary dysmenorrhea (PD) or secondary dysmenorrhea (Proctor and Farquhar, 2006). PD is characterized by pain during the menstrual cycle in the absence of an identifiable cause, and its prevalence ranges from 45% to 95% (Iacovides et al., 2015; Ferries-Rowe et al., 2020). During menstruation, patients with PD often experience abdominal cramping, backache, bloating, headache, nausea, and, in severe cases, syncope (Ma et al., 2021). These symptoms are clearly associated with absenteeism and reduced performance at work or school (Savvas and Brooks, 1991; Dawood, 2006). Recent studies have demonstrated that PD is closely related to indicators such as prostaglandins (PGs), estrogen (E2), progesterone (PG), and NO. Among these, PGF_2α_ is positively correlated with PD, whereas PGE_2_ can induce uterine smooth muscle relaxation (Chan and Hill, 1978; He and Zhou, 2023). E2 and PG primarily regulate the level of PGF_2α_, which in turn contributes to the onset of dysmenorrhea (Wang, 2018). NO is an endogenous regulatory molecule involved in various physiological processes. It dilates blood vessels, relaxes smooth muscle, and inhibits platelet aggregation, thereby alleviating PD symptoms (Sun and Wang, 2012). However, the pathophysiology of PD remains complex, and its treatment mechanisms require further investigation.

Currently, in clinics, cyclooxygenase inhibitors, which belong to the class of non-steroidal anti-inflammatory drugs, such as aspirin, ibuprofen, and naproxen sodium, are commonly prescribed to manage PD. These drugs can relieve pain, but are associated with significant side effects. For instance, non-steroidal anti-inflammatory drugs may cause gastrointestinal complications, acute liver dysfunction and even acute renal failure (Savvas and Brooks, 1991; Gutthann et al., 1996; Ma et al., 2021). Thus, the development of safer and more effective treatment options for PD is essential for improving women’s health.

Traditional Chinese medicine (TCM), a long-standing medical system used in China for thousands of years, is characterized by its multicomponent and multitarget properties (Zhang et al., 2022). Compared to chemical drugs, TCM offers numerous advantages, including minimal side effects, low toxicity, and excellent tolerability (Tang et al., 2023). Classic TCM dosage forms, include decoctions, pills, powders, concentrated decoctions, ointments, plasters, syrups, medicinal wines, suppositories, and tinctures (Duan et al., 2017). Plasters are modern transdermal drug delivery systems offering benefits, such as low toxicity and side effects, prolonged efficacy, and ease of administration. Our group developed a potential formula for a TCM plaster, “Wentong plaster (WTT),” which comprises Corydalis Rhizoma (YHS), Chuanxiong Rhizoma (CX), Salviae Miltiorrhizae Radix Et Rhizoma (DS), Cyperi Rhizoma (XF), Cinnamomi Cortex (RG), and Euodiae Fructus (WZY). Clinical evidence has shown that WTT significantly relieves the pain in patients during menstruation. However, the mechanisms by which WTT treats PD have not yet been clearly elucidated.

Transcriptomics, which can establish biomarkers or pathways in molecular targets, is an emerging tool for studying the expression of RNA transcripts in organisms (VanGilder et al., 2012; Liu et al., 2024). Transcriptomics is commonly used to study the effects and molecular mechanisms of herbal medicines (Zhang et al., 2024). Analyzing the changes in the levels of targets can uncover relevant pathways for targeted therapy. Network pharmacology is a powerful approach that integrates systems biology, computational biology, and experimental techniques across various disciplines (Li et al., 2023; Su et al., 2025; Zheng et al., 2025). It has been successfully applied to screen the material basis and molecular mechanisms of herbal medicines based on the establishment and analysis of topology networks (Li, 2021). The combination of metabolomics and network pharmacology helps evaluate therapeutic effectiveness and explore mechanisms, thereby improving the credibility and acceptance of herbal medicines.

The major aim of this study was to establish a PD rat model, and intervene using the WTT. The effects of the WTT on PD were evaluated based on the levels of E2, NO, and PG. Subsequently, UPLC-Q-Exactive Orbitrap-MS was used to screen the blood-entry constituents. Network pharmacology and transcriptomics were used to reveal the potential mechanisms of WTT in PD. Finally, enzyme-linked immunosorbent assay (ELISA) and western blotting (WB) techniques were used to determine the expression of the relevant targets. This study provides a reference for research on the mechanism of WTT in PD, and offers a scientific implication for WTT in PD in the clinic.

## 2 Materials and methods

### 2.1 Preparation of WTT

The WTT consisted of YHS, CX, DS, XF, RG, and WZY. Briefly, in the first step, 40 g of YHS and 30 g of DS were extracted using 60% ethanol for 1.5 h at a liquid-to-material ratio of 8:1, and concentrated into a solution with a density of 1.25–1.3. In the second step, 18 g of RG, 30 g of CX, 24 g of XF, 10 g of WZY and 20 g of fresh ginger were extracted using steam distillation for 5 h at a liquid to material ratio of 8:1 to obtain the total volatile oil. In the third step, the decoction dregs collected from the forementioned process were extracted using deionized water for 1.5 h at a liquid-to-material ratio of 8:1. Meanwhile, the solution was mixed with the extract from the second step, and concentrated into a solution with a density of 1.06–1.10. Subsequently, in the fourth step, ethanol was added to the solution to achieve a final alcohol concentration of 60%, and the mixture was stored at 4°C. After 24 h, in the fifth step, the solution was concentrated into solution with a density of 1.25–1.30, and mixed with the solution prepared in the first step.

Subsequently, 4.6 g of NP-700 was dissolved in 32 g of glycerol, and aluminum glycinate, total volatile oil and azone were added to the mixture, and named Phase A. Separately, 0.45 g of PVA was dissolved in deionized water, and 3 g of PVP K-90 was added, stirred well, and subjected to ultrasonic treatment (250 W, 33 kHz) for 15 min. Then, 4 g of colloidal silica was added, and stirred thoroughly to obtain Phase B.

Phases A and B were gradually mixed while stirring slowly in a 60°C water bath until fully combined. Subsequently, the solution prepared in the fifth step was added, and stirred continuously in a 60°C water bath until homogeneity was achieved, followed by ultrasonic treatment for 20 min. Finally, the mixture was spread onto a nonwoven fabric backing at 45°C, with a thickness of approximately 2.0 mm, and dried at 40°C for 12 h.

### 2.2 Animal treatment

All experimental methodologies and protocols were subjected to a rigorous review and were formally endorsed by the Institutional Animal Ethics Committee at Shandong Academy of Chinese Medicine, situated in Jinan, China, under the authorization number SDZYY20210925001. The 8-week-old female Sprague–Dawley (SD) rats used in the present study were provided by Jinan Pengyue Laboratory Animal Breeding Co., Ltd. The rats reside in a controlled environment characterized by a temperature range of 22°C–25°C, accompanied by a relative humidity level between 50%–60%, and subject to regular 12 h cycles of light and darkness, ensuring optimal living conditions for the animals.

A rat model of PD characterized by cold coagulation and blood stasis syndrome was induced using cold stimulation coupled with estradiol (E2), and oxytocin. Briefly, 40 SD rats were adaptively fed for 6 days randomly divided into four groups: control group (CG), model group (PD), Wenjing Zhitong plaster (WJZT) treatment group, and WTT treatment group. The rats in the PD, WJZT, and WTT groups, were placed on ice blocks for 20 min daily throughout the experiment. E2 (0.8 mg/rat) was administered via subcutaneous injection on the seventh and eighteenth days. E2 (0.4 mg/rat) was administered daily via subcutaneous injection from the eighth day to the seventeenth day. On the final day, after E2 was injected for 1 h, oxytocin at a dose of 2 IU/rat was administered subcutaneously. Starting on the twelfth day of modeling, CG and PD groups were treated with a blank plaster, whereas the WJZT and WTT groups were treated with WJZT and WTTs, respectively, applied to the Shenque acupoint on the rat abdomen. Treatment was administered once daily for six consecutive days.

Subsequently, a full-field laser perfusion imager coupled with a single CCD imaging technology (FLPI, Moore, United Kingdom) was used to observe blood flow within the analysis areas of the paws, sublingual region, and uterine tissue of the rats. In addition, the writhing response of each group of female rats was evaluated within 30 min according to the behavioral scoring criteria proposed by Schmauss and Yaksh (1984). The latency to the first writhing response after administration and total number of writhing events within 30 min were recorded. Finally, the rats were sacrificed under isoflurane inhalation anesthesia, and their plasma and uterine tissues were collected and stored at −80°C for further analysis.

### 2.3 ELISA

To evaluate the effect of WTT on rats with PD, specific ELISA kits were used following the manufacturer’s instructions to detect the content of PGE_2_ (Lot number: 202308, Suzhou Xunjie Biotechnology Co., Ltd, China), PGF_2*α*_ (Lot number: 202308, Suzhou Xunjie Biotechnology Co., Ltd, China), E2 (Lot number: 9680016083, Wuhan Self Love Botaike Biotechnology Co., Ltd, China), PG (Lot number: 9680014085, Wuhan Self Love Botaike Biotechnology Co., Ltd, China), and NO (Lot number: 202308, Suzhou Xunjie Biotechnology Co., Ltd, China) in serum, and content of Cxcl6 (Lot number: RA22642, Wuhan Beiyinlai Biotechnology Co., Ltd, China), Ccl20 (Lot number: RA20605, Wuhan Beiyinlai Biotechnology Co., Ltd, China), Lcn2 (Lot number: RA20783, Wuhan Beiyinlai Biotechnology Co., Ltd, China), and IL-17 (Lot number: RA20117, Wuhan Beiyinlai Biotechnology Co., Ltd, China) in uterine tissue.

### 2.4 Histopathological analysis

Pathological changes in rat uterine tissues were observed using hematoxylin and eosin (H&E) staining. First, the specimens were fixed in paraformaldehyde solution. After 24 h, alcohol was used to dehydrate the uterine tissues which were then cleared with xylene. Finally, sections with a thickness of 4 μm were prepared for observation.

Pathological changes were evaluated using the McGuigan pathological scoring system. The score was influenced by multiple indicators, including the proliferation of uterine mucosal epithelial cells, hypercolumnar changes in these cells, hyperplasia of mucosal glands, and increased secretion. The score increased with the number and severity of lesions.

### 2.5 Coagulation test

To assess the coagulation function of rats, the levels of coagulation indicators, such as prothrombin time (PT), fibrinogen (FIB), activated partial thromboplastin time (APTT) and thrombin time (TT), were detected using a semi-automatic coagulation analyzer (PUN-2048B, Beijing Pulang New Technology Co., Ltd., China) coupled with the respective kits: PT Assay Kit (freeze-dried, coagulation method, Lot number: 105510, Shanghai Sun Biotechnology Co., Ltd, China), FIB Assay Kit (freeze-dried, coagulation method, Lot number: 1322151, Shanghai Sun Biotechnology Co., Ltd, China), APTT Assay Kit (ellagic acid, fixed-time method, Lot number: 1122651, Shanghai Sun Biotechnology Co., Ltd, China), and TT Assay Kit (freeze-dried, coagulation method, Lot number: 121337, Shanghai Sun Biotechnology Co., Ltd, China).

### 2.6 UPLC-Q-Exactive Orbitrap-MS analysis

Using plasma as the sample, UPLC-Q-Exactive Orbitrap-MS (Thermo Fisher Scientific company, United States) was employed to screen for blood-entry constituents. Specifically, 500 μL of plasma was mixed with 2 mL of a methanol-acetonitrile solution (1:1, v/v). After mixing, the solution was centrifuged at 10,000 rpm for 15 min. Subsequently, 1.5 mL of the supernatant was collected and dried under nitrogen at 30°C. After drying, the sample was redissolved in 100 μL of 70% methanol solution, and centrifuged at 12,000 rpm for 10 min. Finally, the supernatant was collected for analysis using UPLC-Q-Exactive Orbitrap-MS. Additionally, WTT (0.5 g) was mixed with 70% methanol (50 mL), and sonicated for 30 min. Additionally, 70% methanol was used to compensate for any losses. The solution was filtered through a 0.22 μm microporous membrane before analysis.

Subsequently, 5 μL of the sample was injected and loaded onto a Thermo Scientific Syncronis C_18_ chromatography column (100 mm × 2.1 mm, 1.7 μm), and eluted with acetonitrile (A) and 0.1% formic acid (B) as the gradient elution: 0 min, 10%A, 3 min, 40%A, 10 min, 40%A, 12 min, 55%A, 22 min, 60%A, 32 min, 90%A, 33 min, 90%A, and 36 min, 10%A. The flow rate was set to 0.3 mL/min.

After separation, the compounds were analyzed using a Q-Exactive Orbitrap Plus high-resolution mass spectrometer. Mass spectrometric analysis was conducted using a heated electrospray ionization source in both positive and negative ion modes. The scan mode was set to Full MS/dd-MS^2^, with a scan range of 80–1,200 m/z. The resolution for full scan MS was 70,000, whereas that for MS^2^ was 17,500. The capillary voltage was set to 3500 V in positive ion mode and 3200 V in negative ion mode. The capillary temperature was maintained at 350°C, and the source temperature was set to 320°C. Both the sheath and auxiliary gases were nitrogen, with sheath gas flow rate of 40 arb and an auxiliary gas flow rate of 10 arb. The S-Lens RF Level was set at 50. For collision-induced dissociation, stepwise collision energies were set at 15, 30, and 45 V.

The acquired data was processed using TraceFinder 5.0 software. Subsequently, the chemical components were identified based on the TCMSP, ChemicalBook and PubChem databases and their cleavage rules.

### 2.7 Network pharmacology analysis

A network pharmacology approach was used to explore the therapeutic mechanisms of WTT in PD. The potential targets of the blood-entry components were predicted using SwissTargetPrediction (http://swisstargetprediction.ch/) and Super-PRED (https://prediction.charite.de/index.php) databases. Potential targets were selected using the Venn platform (https://bioinfogp.cnb.csic.es/tools/venny/index.html). The protein-protein interaction (PPI) network was constructed using the STRING platform (https://cn.string-db.org/), and key targets were identified based on the degree values in the topology network. KEGG enrichment analysis was conducted using the DAVID database (https://david.ncifcrf.gov/). Finally, a “components-targets-pathways” network was established to reveal the potential mechanism of WTT in treating PD.

### 2.8 Transcriptomics

Total RNA was extracted from rat uterine tissues using TRIzol reagent (Life Technologies, Carlsbad, CA, United States). An Agilent Bioanalyzer 4150 system (Agilent Technologies) was used to assess the integrity of the extracted RNA. Sequencing was performed using an Illumina NovaSeq 6000 or MGISEQ-T7 instrument. After sequencing, the raw data were screened to eliminate low-quality segments, were defined as those in which over 60% of a read consisted of bases with a quality score not exceeding 25. The remaining data were mapped using HISAT2 software (http://daehwankimlab.github.io/hisat2/). Differentially expressed genes (DEGs) were identified based on the thresholds, |log_2_FC| > 1 and *P*adj <0.05.

### 2.9 WB

First, the uterine were ground in liquid nitrogen. Subsequently, 10 mg of the sample was mixed with 100 μL of PBS, and lysed on ice for 30 min. The mixture was centrifuged at 12,000 rpm for 15 min (at 4°C). The bicinchoninic acid method was used to determine the concentration of protein samples. Subsequently, the protein samples were diluted with 5 × sodium dodecyl sulfate-polyacrylamide gel electrophoresis protein loading buffer and PBS to a uniform concentration, and heated at 100°C for 10 min to fully denature the protein. Total protein was subjected to sodium dodecyl sulfate-polyacrylamide gel electrophoresis and transferred onto a PVDF membrane. The membranes were blocked with 5% BSA for 1 h before incubation with specific primary antibodies at 4°C overnight. Subsequently, the goat anti-rabbit/mouse HRP secondary antibody was diluted 1:5000 using the blocking solution, and added to the incubation box containing the PVDF membrane, which was incubated with slow shaking at room temperature for 1 h. After the incubation, the secondary antibody was discarded, and the PVDF membrane was washed three to five times with 1× TBST on a shaker, with each wash lasting 5 min. Finally, the protein expression was analyzed using chemiluminescence imaging instrument.

### 2.10 Statistical analysis

All statistical analyses were performed using GraphPad Prism 9, and the data are presented as the mean ± standard deviation (SD). One-way ANOVA was used to analyze differences among groups. Two-sided p values were considered, and p < 0.05 was regarded as statistically significant. Different levels of significance were annotated as follows: compared with CG group, *p < 0.05, **p < 0.01, ***p < 0.001; ****p < 0.0001; compared with PD group, #*p* < 0.05, ##*p* < 0.01; ###*p* < 0.001; ####*p* < 0.0001.

## 3 Results

### 3.1 WTT improved blood flow and reduced the writhing response

**FIGURE 1:**
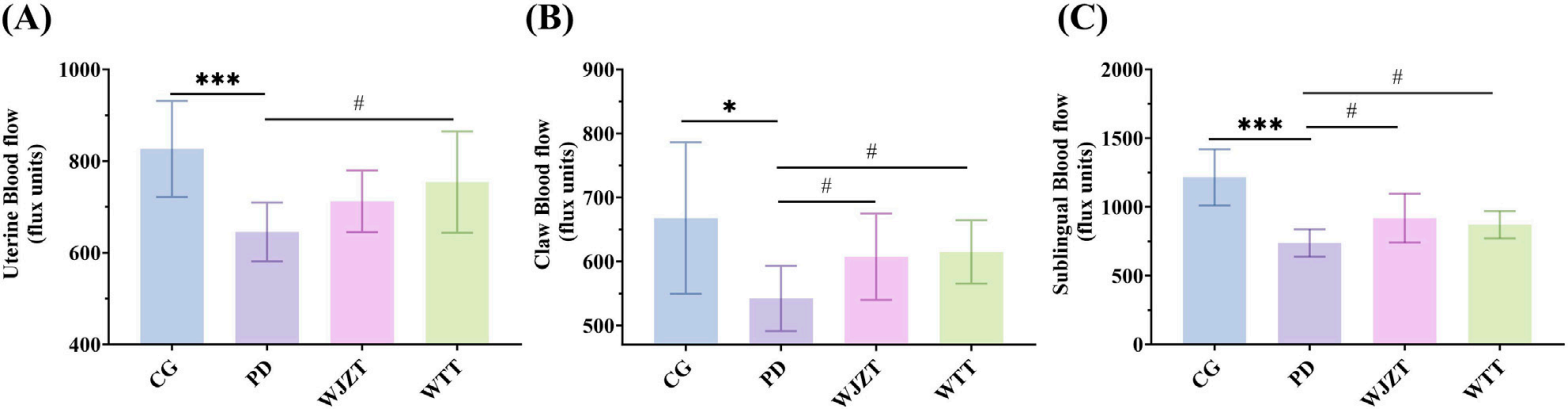
Blood flow in uterine **(A)**, claw **(B)**, and sublingual **(C)**. Compared with CG group: *p < 0.05, **p < 0.01, ***p < 0.001, ****p < 0.0001; compared with PD group: #*p* < 0.05, ##*p* < 0.01, ###*p* < 0.001, ####*p* < 0.0001. CG, control group; PD, primary dysmenorrhea model group; WJZT, Wenjing Zhitong plaster treatment group; WTT, Wentong plaster treatment group.

Owing to the internal microcirculation of blood in the body and abundant blood vessels distributed in the sublingual region, the sublingual area can reflect the state of blood flow (Jin, 2022). Furthermore, a study demonstrated that poor blood circulation was associated with reduced blood flow in the claws and sublingual region (Cui, 2022). Thus, blood flow in the claws, sublingual region, and uterine tissues of the rats was observed using laser speckle contrast imaging (Supplementary Figures S1–S3). As shown in Figure 1, rats in the PD group exhibited lower blood flow in the uterine, claw, and sublingual areas than that of those in the CG group, suggesting that PD characterized by cold coagulation and blood stasis syndrome was successfully established. The WJZT and WTT groups exhibited significantly improvement in the blood flow.

As shown in Supplementary Table S1, compared with observations in the CG group, the latency of the writhing response in rats in the PD group was significantly shortened, and the number of writhing reactions within 30 min was significantly increased, indicating that the strong uterine contractions caused by oxytocin acting on the uterus can lead to abdominal pain and writhing reactions in rats, suggesting that PD characterized by cold coagulation and blood stasis syndrome was successfully established. Compared with that in the PD group, the latency of the writhing reaction in rats in the WTT group was significantly prolonged (*p* < 0.05), and the number of writhing responses was significantly reduced (*p* < 0.01), suggesting that WTT has a significant analgesic effect on PD in rats.

### 3.2 Effect of WTT on the levels of indicators in serum and uterine

**FIGURE 2:**
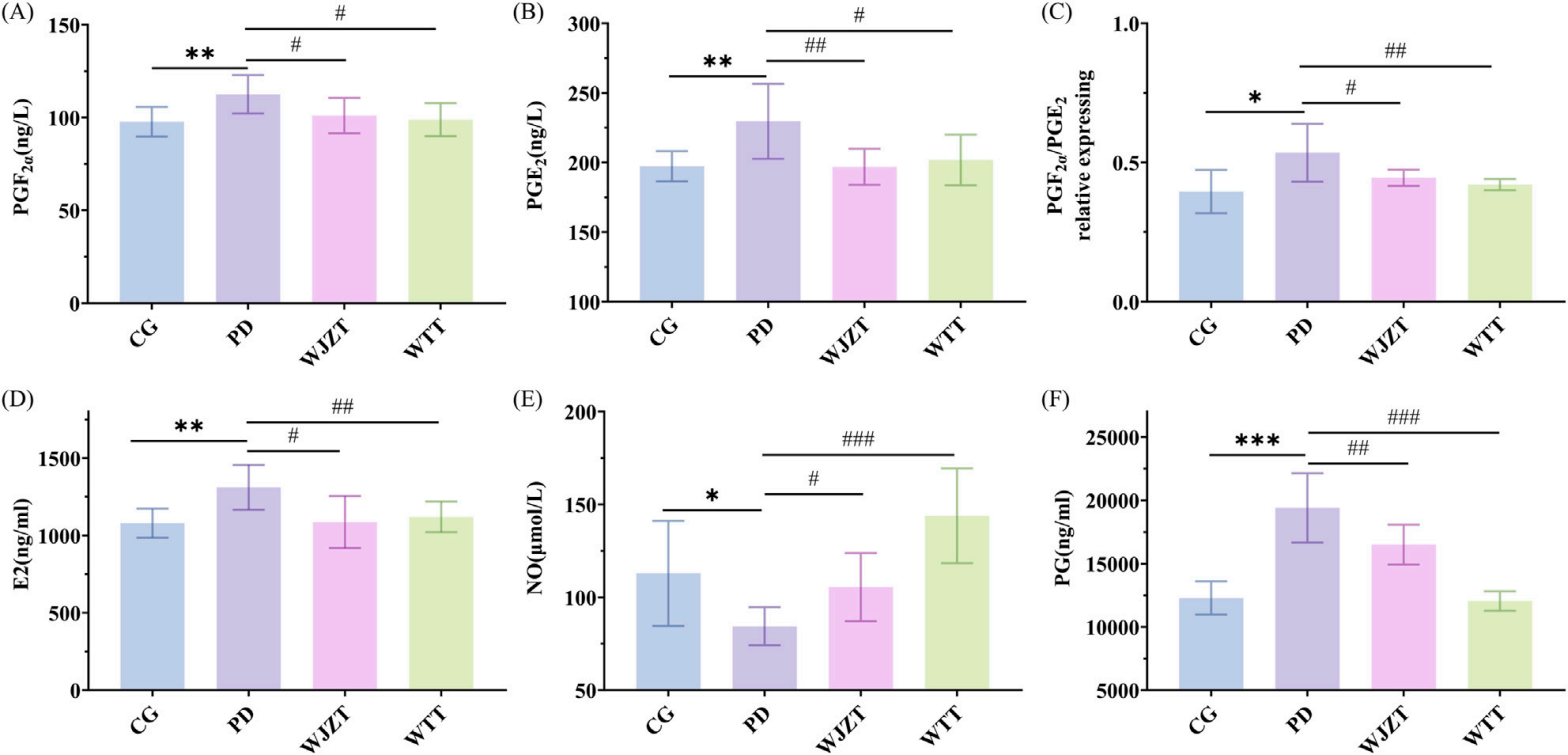
Effect of WTT on levels of PGF_2α_ **(A)**, PGE_2_ **(B)**, PGF_2α_/PGE_2_ relative expression **(C)**, E2 **(D)**, NO **(E)**, and PG **(F)**. Compared with CG group: *p < 0.05, **p < 0.01, ***p < 0.001, ****p < 0.0001; compared with PD group: #*p* < 0.05, ##*p* < 0.01, ###*p* < 0.001, ####*p* < 0.0001. CG, control group; PD, primary dysmenorrhea model group; WJZT, Wenjing Zhitong plaster treatment group; WTT, Wentong plaster treatment group; E2, estrogen; PG, progesterone.

Compared with those in the CG group, the PGF_2*α*_ and PGE_2_ levels in the plasma of rats in the PD group were significantly increased (*p* < 0.05). Compared with those in the PD group, the WTT group exhibited significantly decreased PGF_2*α*_ levels (*p* < 0.01) and significantly decreased PGE_2_ levels (*p* < 0.05) in plasma, with an evidently reduced PGF_2*α*_/PGE_2_ ratio (*p* < 0.05) (Figures 2A–C). These results indicate that WTT can reduce PGF_2*α*_ content and increase PGE_2_ content in rats with PD, thereby alleviating smooth muscle contraction and producing analgesic effects.

As shown in Figures 2D–F, the levels of E2, NO, and PG were measured. Compared with those in the CG group, there was a significant increase in the levels of E2 and PG in the PD group (*p* < 0.01), and a significant decrease in the level of NO (*p* < 0.05). Compared with observations in the PD group, WTT significantly reduced E2 and PG levels (*p* < 0.01) and significantly upregulated NO levels in rats with PD (*p* < 0.01).

### 3.3 Histopathological analysis

**FIGURE 3:**
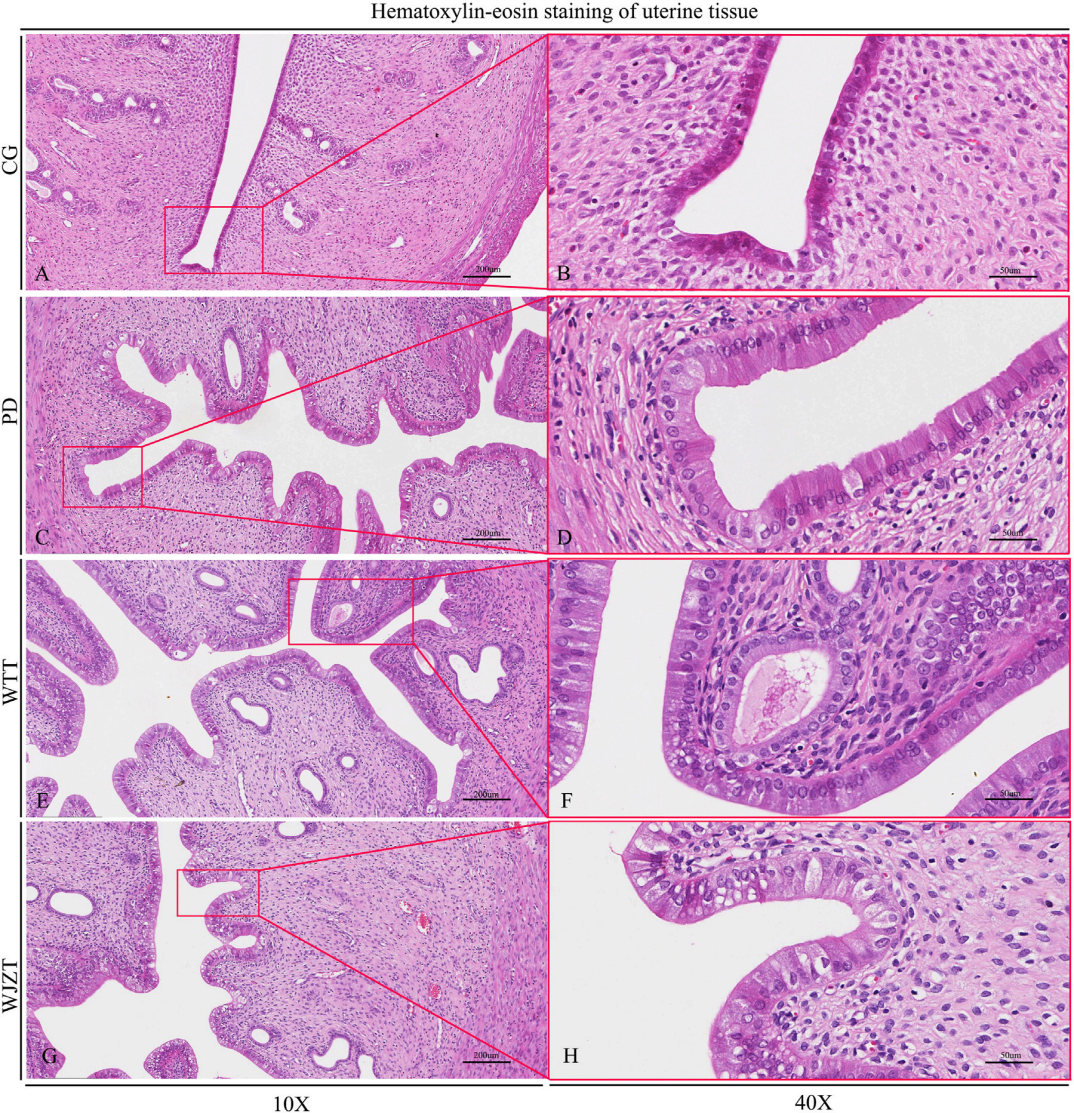
Histopathological analysis of rats. CG, control group, 10× **(A)**, 40× **(B)**; PD, primary dysmenorrhea model group, 10× **(C)**, 40× **(D)**; WJZT, Wenjing Zhitong plaster treatment group, 10× **(E)**, 40× **(F)**; WTT, Wentong plaster treatment group, 10× **(G)**, 40× **(H)**.

The pathological changes in rat uterine tissue are shown in Figure 3. The uterine tissue structure in the CG group was intact and clear, without hyperplasia or inflammatory cell infiltration. The uterine glands were normal, with no signs of hyperplasia or inflammation. A small number of eosinophils were present in the lamina propria, and the mucosal layer had normal thickness, without edema or bleeding. In the PD group, endometrial epithelial cells exhibited hyperplasia and tall columnar changes. The number of glands in the endometrium increased, with a dilated glandular lumen and active secretory function. Rats in the WTT and WJZT groups showed similar changes to those in the PD group but to a lesser extent.

As shown in Supplementary Table S2, the pathological scores of rat uterine tissue were calculated using McGuigan’s criteria. Compared with that in the CG group, the total score in the PD group increased significantly (*p* < 0.01), with statistically significant differences in all four parameters: hyperplasia of uterine mucosal epithelial cells, tall columnar changes in epithelial cells, hyperplasia of mucosal glands, and increased secretion. Compared with those of the PD group, WTT group showed significantly lower total scores and reductions in all four parameters (*p* < 0.05). Notably, the decreases in hyperplasia of uterine mucosal epithelial cells, tall columnar changes, and increased secretion were extremely significant (*p* < 0.01). The WJZT group showed similar improvements, with a highly significant reduction in epithelial hyperplasia (*p* < 0.01) and significant reductions in columnar changes, glandular hyperplasia, and secretion (*p* < 0.05).

### 3.4 Coagulation test

**FIGURE 4:**
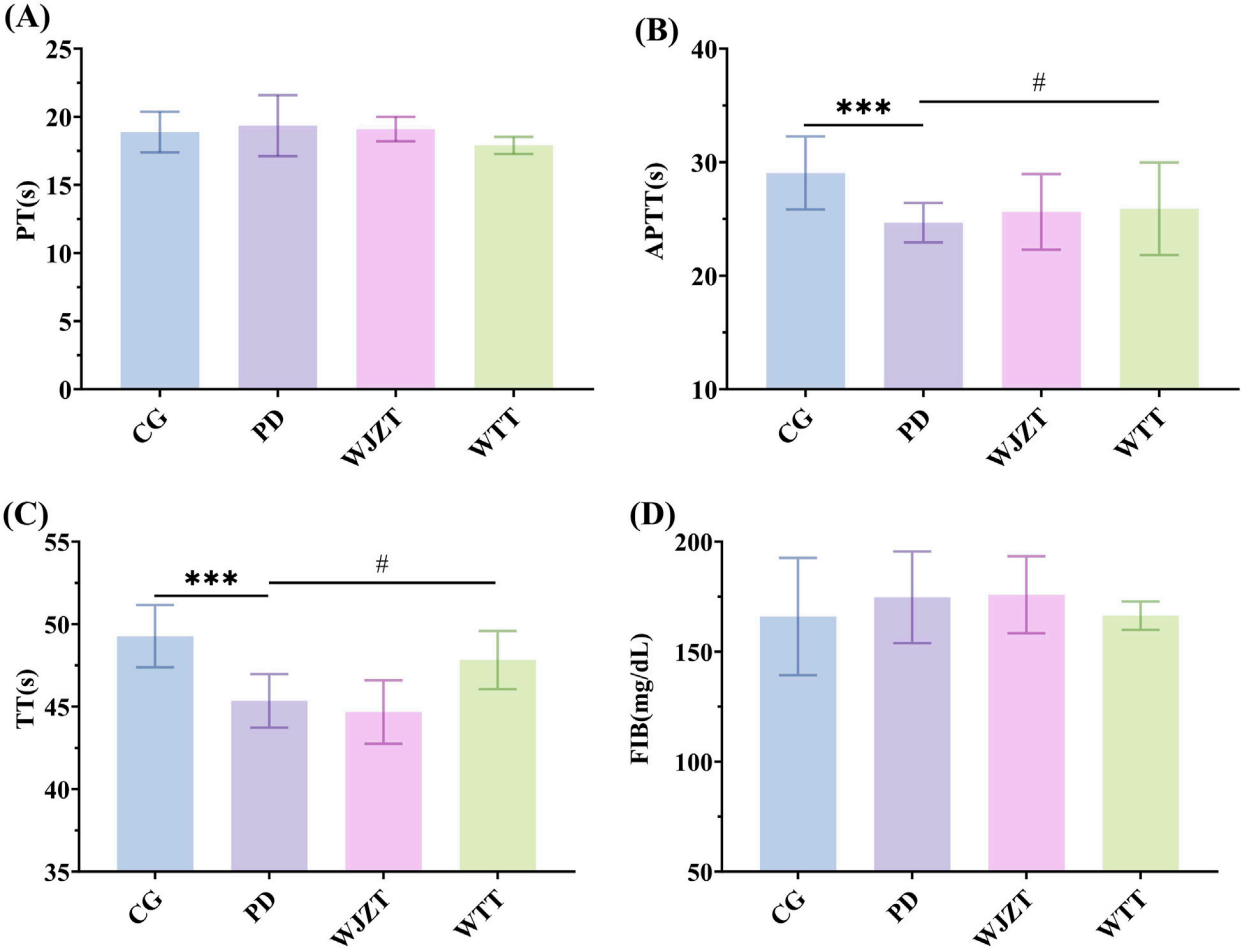
Effects of WTT on PT **(A)**, APTT **(B)**, TT **(C)**, and FIB **(D)**. Compared with CG group: *p < 0.05, **p < 0.01, ***p < 0.001, ****p < 0.0001; compared with PD group: #*p* < 0.05, ##*p* < 0.01, ###*p* < 0.001, ####*p* < 0.0001. CG, control group; PD, primary dysmenorrhea model group; WJZT, Wenjing Zhitong plaster treatment group; WTT, Wentong plaster treatment group; PT, prothrombin time; FIB, fibrinogen, APTT, activated partial thromboplastin time; TT, thrombin time.

As shown in Figure 4, compared with those in the CG group, rats in the PD group showed significant reductions in TT and APTT (*p* < 0.01), whereas there were no significant differences in FIB and PT, indicating increased coagulation factor activity and abnormal coagulation function. Compared with those in the PD group, rats in the WTT group exhibited a significant increase in TT (*p* < 0.05). Meanwhile, WTT downregulated PT and upregulated APTT, the differences were not statistically significant. These results suggest that WTT can partially alleviate the hypercoagulable state in rats with PD.

### 3.5 Blood-entry components analysis

UPLC-Q-Exactive Orbitrap-MS was used to analyze the rat plasma containing the medicine (Supplementary Figure S4A, S5A). Based on retention times, *m/z* and fragments, 175 blood-entry components were identified, including 49 original blood-entry components and 126 metabolites (Supplementary Table S3). These original blood-entry components were mainly divided into 11 organic acid compounds, 6 alkaloids, 12 terpenes, 2 saponins, 1 amine, 1 alcohol, 1 phenol, 3 quinones, 1 retinoid, 1 phosphatidylcholine, 1 sphingolipid, 2 aldehydes, 5 ketones, 1 amide, and 1 coumarin compound. Metabolites were generated through oxidation, methylation, glucuronidation, reduction reactions, and sulfonation reactions.

The WTT contained a primary ion of cinnamaldehyde with an *m/z* of 174.0915 [(M + ACN + H)^+^]. Based on comparison with the chromatogram of the WTT solution and database searches, P4 was identified as cinnamaldehyde. The molecular ion peaks of M12 and M31 were *m/z* 206.0815 [(M + ACN + H)^+^] and 161.0599 [(M + H-H2O)^+^], respectively. Secondary mass spectrum fragments included *m/z* 155.0938, 140.0703, 124.0757, and 96.0811. This fragmentation pattern was consistent with cinnamaldehyde, suggesting that M12 is a metabolic product of cinnamaldehyde. The molecular mass difference of 32 Da between M12 and cinnamaldehyde indicated that cinnamaldehyde underwent Phase I metabolism *in vivo*, resulting in the formation of M12, a hydroxycinnamic acid derivative. Additionally, M31, an oxidized and methylated metabolite, was identified as 4-methoxycinnamic acid. The proposed metabolic pathway is shown in Supplementary Figure S6A.

The WTT sample contained dehydroabietic acid with a primary ion at *m/z* 301.2164 [(M + H-H2O)^+^]. Based on comparisons with the chromatogram of the WTT solution and database searches, the bloodstream component was identified as dehydroabietic acid (P31). The molecular ion peak of M61 was *m/z* 255.2110 [(M + H-H2O)^+^], with secondary mass spectrum fragments *m/z* 69.0706, 197.1326, and 199.1484. The fragmentation pattern was consistent with that of dehydroabietic acid, suggesting that M61 is a metabolic product of dehydroabietic acid. The molecular mass difference of 32 Da between M61 and dehydroabietic acid indicated that dehydroabietic acid underwent Phase I metabolism *in vivo*, and was reduced to 18-Norabieta-8,11,13-trien-4-ol. The proposed metabolic pathway is shown in Supplementary Figure S6B.

The molecular ion peak of M38 was *m/z* 283.1186 [(M + FA-H)^−^]. The characteristic secondary fragment ions included *m/z* 265.1232 and 247.1124, with a fragmentation pattern consistent with that of osthole A. Therefore, we hypothesized that the precursor ion was osthole A. The molecular mass difference of 176 Da between M38 and osthole A indicated that M38 was derived from the oxidation, methylation, and other Phase I metabolic reactions of osthole A, resulting in the formation of 6-hydroxy-7-methoxydihydroligustilide. The proposed metabolic pathway is shown in Supplementary Figure S6C.

### 3.6 Network pharmacology analysis

**FIGURE 5:**
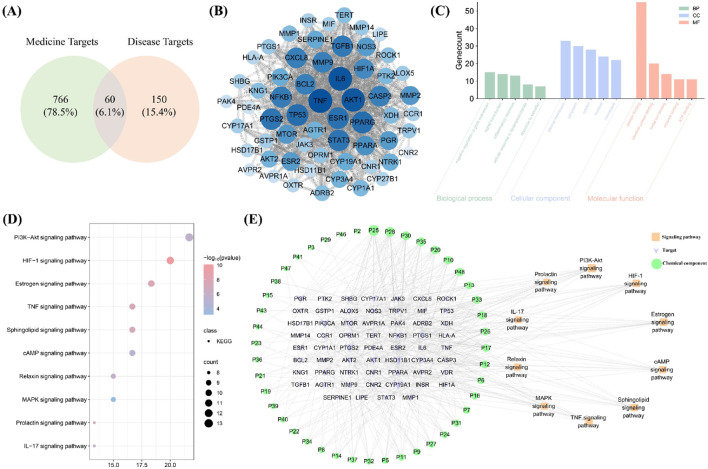
Network pharmacology analysis: Venny plot **(A)**; PPI network **(B)**; GO enrichment analysis **(C)**; KEGG enrichment analysis **(D)**; “Components Targets-Pathways” network **(E)**.

Network pharmacology analysis identified 826 medicine targets related to 49 blood-entry components. In addition, 210 disease targets (relevance score>5) associated with PD were identified. Sixty potential targets were determined (Figure 5A). As shown in Figure 5B, the PPI network analysis revealed TNF, IL6 and AKT1 as key targets within the topological network, suggesting that blood-entry components may primarily act on these key targets, thereby regulating other downstream targets. Furthermore, GO enrichment analysis revealed significant enrichment of potential targets in several biological processes (BP), molecular functions (MF), and cellular components (CC). In the BP category, the most enriched pathways included inflammatory response, cellular response to lipopolysaccharide, and response to estradiol, indicating that these targets may play a key role in inflammation and E2 regulation. For the CC category, potential targets were enriched in plasma membrane, cytoplasm, and cytosol, implying that these genes may be crucial for maintaining cellular structure and function. In the MF category, the enriched terms primarily involved protein binding, identical protein binding, and enzyme binding, suggesting potential involvement of these targets in signal transduction and protein-protein interactions (Figure 5C). KEGG pathway enrichment analysis indicated that HIF-1 signaling pathway, E2 signaling pathway, and IL-17 signaling pathway were important signaling pathways regulated by the blood-entry components of WTT, suggesting that WTT may treat PD by regulating inflammatory responses and E2 levels (Figure 5D). As illustrated in Figure 5E, the topological network comprises 114 nodes and 437 edges. Green, purple and orange nodes represent blood-entry components, targets and pathways, respectively. These findings suggest that the mechanism by which WTT treats PD is complex, reflecting the pharmacological characteristics of multicomponent regulation of multitarget systems.

### 3.7 Transcriptomics analysis

**FIGURE 6:**
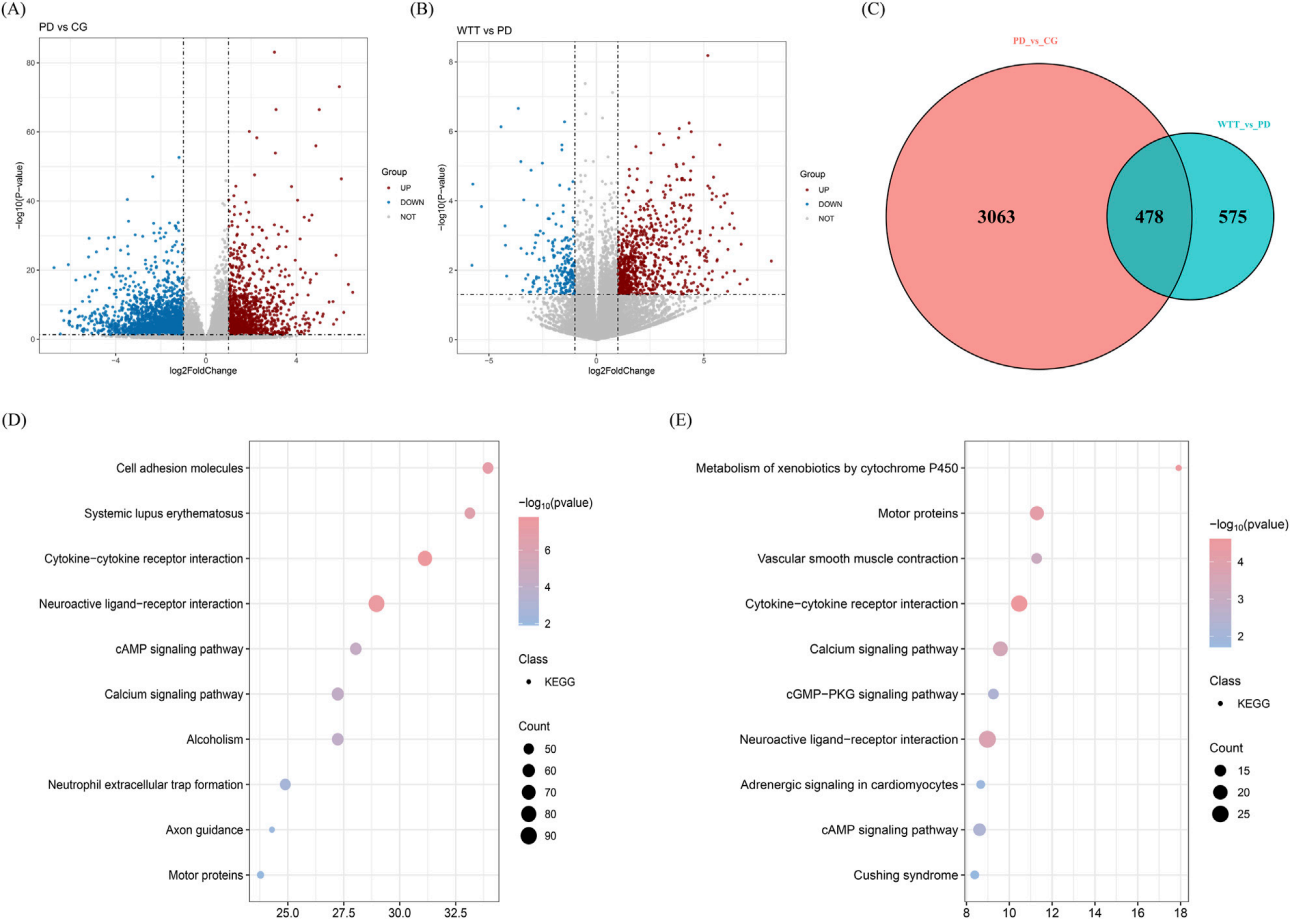
The volcano plot of DEGs screened between PD and CG groups **(A)** and between WTT and PD groups **(B)**; Venny plot **(C)**; The KEGG enrichment analysis of DEGs screened between PD and CG groups **(D)** and between WTT and PD groups **(E)**.

Uterine tissues from CG, PD, and WTT groups were collected to detect RNA and identify differentially expressed genes (DEGs). As shown in Figure 6A, in the comparison between the PD and CG groups, 3,541 DEGs were identified, including 1,270 upregulated and 2,271 downregulated genes. As shown in Figure 6B, in the comparison between the WTT and PD groups, a total of 1,053 DEGs were identified, including 834 upregulated and 219 downregulated genes. Subsequently, 478 DEGs identified by integration (Figure 6C).

To better understand the biological processes of the DEGs involved in the response of WTT to PD treatment. GO and KEGG enrichment analyses were performed. As shown in Supplementary Figure S7A, the DEGs screened in the comparison between the PD and CG groups were mainly enriched in biological processes such as cell periphery, multicellular organismal processes, and molecular transducer activity. As shown in Supplementary Figure S7B, the DEGs screened in the comparison between the WTT and PD groups were mainly enriched in biological processes such as axoneme assembly, axoneme, and minus-end-directed microtubule motor activity. Additionally, as shown in Figure 6D, the KEGG enrichment results suggested that the DEGs screened in the comparison between the PD and CG groups were mainly enriched in signaling pathways, such as neuroactive ligand-receptor interaction, cytokine-cytokine receptor interaction, and the calcium signaling pathway. As shown in Figure 6E, the KEGG enrichment results suggested that the DEGs screened in the comparison between the WTT and PD groups were mainly enriched in signaling pathways, such as neuroactive ligand-receptor interaction, cytokine-cytokine receptor interaction, and the calcium signaling pathway. Among these, neuroactive ligand-receptor interaction showed the highest enrichment of DEGs, and cytokine-cytokine receptor interaction was identified as an important pathway. These results indicate that the DEGs were mainly related to cytokines and their receptor activity.

More importantly, based on the results of animal experiments and network pharmacology analysis, WTT induced therapeutic effects in PD by regulating the inflammatory response and E2 levels. Therefore, signaling pathways directly related to inflammation were selected. For instance, the DEGs screened in the comparison between the WTT and PD groups were significantly enriched in the IL-17 signaling pathway. The DEGs screened in the comparison between the PD and CG groups showed no significant enrichment in the IL-17 signaling pathway. However, multiple genes related to IL-17 signal pathway showed significant differences in the comparison between the PD and CG groups, including Cxcl6, Ccl20 and Lcn2. Therefore, the mechanism of action of WTT is to improve PD by inhibiting the IL-17 signaling pathway mediated by chemokine activity. These indicators were selected for further analysis.

### 3.8 Effect of WTT on the protein expression levels of indicators in uterine tissues

**FIGURE 7:**
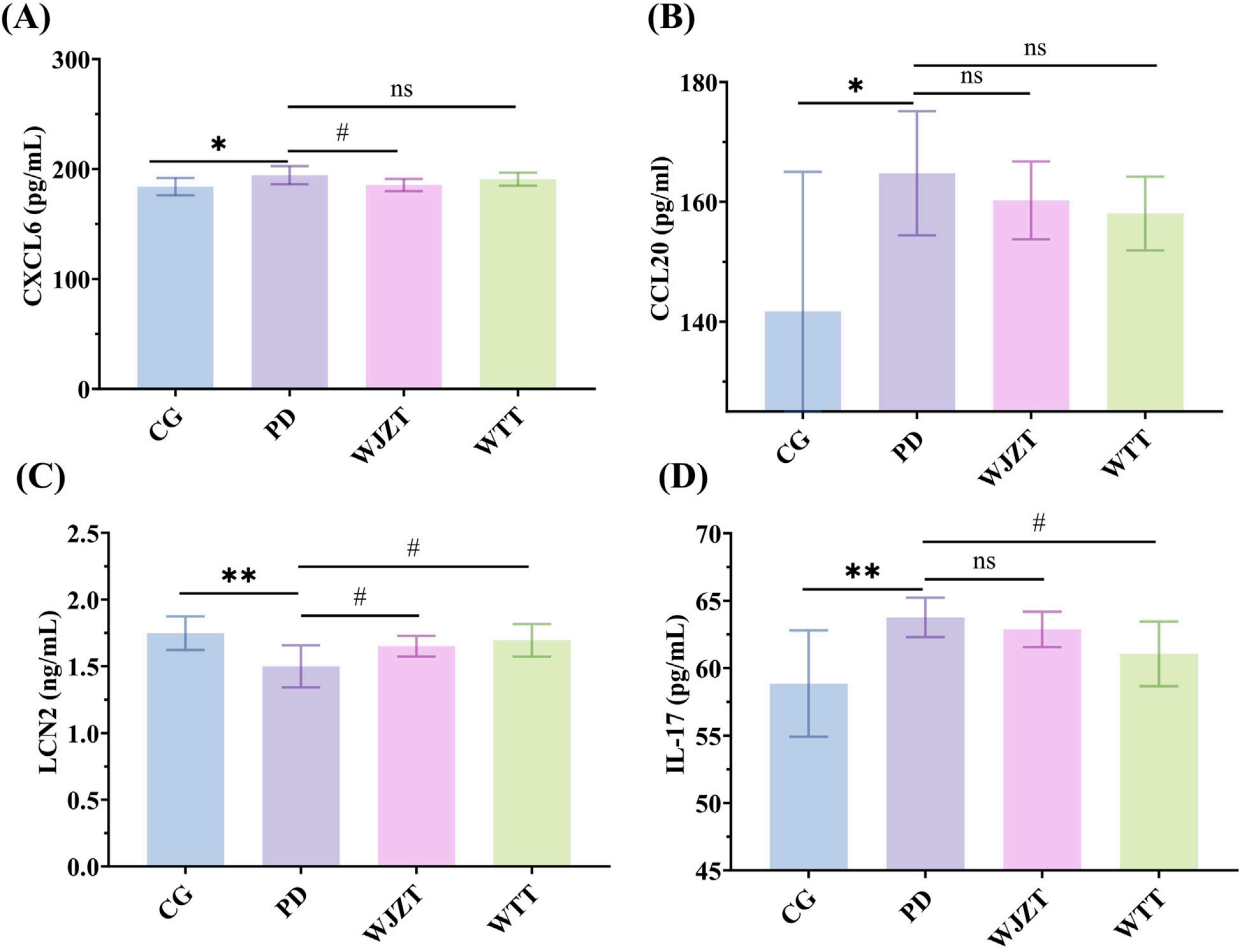
The levels of Cxcl6 **(A)**, Ccl20 **(B)**, Lcn2 **(C)**, and IL-17 **(D)** in uterine tissue of PD rats. Compared with CG group: *: *p* < 0.05, **: *p* < 0.01; ***: *p* < 0.001; ****: *p* < 0.0001; Compared with PD group: #: *p* < 0.05, ##: *p* < 0.01; ###: *p* < 0.001; ####: *p* < 0.0001.

To further reveal the mechanism of WTT in PD, the levels of Cxcl6, Ccl20, Lcn2, and IL-17 in the uterine tissues of rats with PD were determined. As shown in Figure 7A, Cxcl6 levels were increased in the PD group; however, WTT did not significantly downregulate Cxcl6 levels. Compared with that in the CG group, the level of Ccl20 was increased in the PD group, whereas WTT did not significantly downregulate its expression (Figure 7B). Furthermore, as illustrated in Figure 7C, decreased expression of Lcn2 was observed in the PD group, whereas an increased level of Lcn2 was detected in the WTT group with a significant difference between the WTT and PD groups. Compared with that in the CG group, the level of IL-17 increased in the PD group, but decreased in the WTT group (Figure 7D).

**FIGURE 8:**
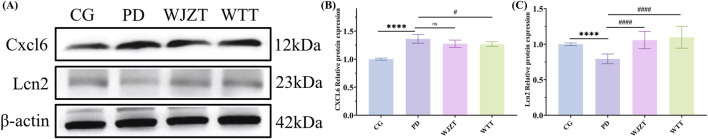
Effect of WTT on Cxcl6 and Lcn2 protein levels in PD rats determined by WB **(A)**, and the ratio of Cxcl6 **(B)** and Lcn2 **(C)** to *β*-actin was calculated. Compared with CG group: *: *p* < 0.05, **: *p* < 0.01; ***: *p* < 0.001; ****: *p* < 0.0001; Compared with PD group: #: *p* < 0.05, ##: *p* < 0.01; ###: *p* < 0.001; ####: *p* < 0.0001.

Subsequently, the protein levels of Cxcl6 and Lcn2 were further evaluated by WB (Figure 8). Cxcl6 levels were higher in the PD group than in the CG group, whereas the Lcn2 levels were lower in the PD group than in the CG group. In the WTT group, the protein levels of Cxcl6 were lower than those in the PD group, whereas the protein level of Lcn2 was higher than in the CG group. These results suggest that WTT could alleviate PD by regulating the levels of IL-17, Cxcl6, and Lcn2 and further downregulating the IL-17 signaling pathway to inhibit the inflammatory response. However, based on the network pharmacology results, these indicators were not directly regulated by blood-entry compounds. Interestingly, by establishing the “blood-entry compounds–direct/indirect targets–IL-17 signaling pathway” topological network, it was found that blood-entry compounds could regulate the expression of direct targets, then influence the levels of indirect targets, ultimately downregulating the IL-17 signaling pathway (Supplementary Figure S8).

## 4 Discussion

PD has become a common gynecological condition, affecting between 45%–95% of menstruating women (Iacovides et al., 2015). PD usually causes lower abdominal and back pain, thereby influencing attendance and decreasing efficacy at work and school (Ferries-Rowe et al., 2020). Over 15% of women with PD experience absenteeism from work, school, and other activities (Dawood, 2006; Ju et al., 2014). However, PD is a complex pathological progression condition closely related to the neuroendocrine-immune network (Ma et al., 2021). Recently, several chemical drugs have been developed for the treatment of PD. However, these drugs are often associated with adverse effects. TCM is a powerful tool for maintaining health in China and is characterized by multicomponent, multitarget properties [8]. WTT, a TCM plaster developed by our group, is an optimized formula based on the Shaofu Zhuyu decoction. It is composed of YHS, CX, DS, XF, RG, and WZY. A previous study suggested that tetrahydropalmatine, an active substance in YHS, exerts a therapeutic effect on dysmenorrhea caused by endometriosis by intervening in the EGFR/PI3K/AKT signaling pathway (Zhang et al., 2025). CX inhibits the contraction of rat uterine smooth muscle, which is beneficial for treating dysmenorrhea (Zhong et al., 2018). Tanshinone IIA, a major active component, can reduce Bcl-2 levels and increase Bax and Caspase-3 levels in the serum of patients, thereby alleviating dysmenorrhea symptoms (Yang, 2016). XF is commonly used in the treatment of gynecological diseases and exerts analgesic effects by regulating arachidonic acid and linoleic acid metabolism, as well as the biosynthesis of unsaturated fatty acids. Specifically, it decreases the levels of 2-series prostanoids and 4-series leukotrienes, which are proinflammatory, induce platelet aggregation, and cause vasoconstriction. By reducing their levels, XF contributes to the treatment of gynecological conditions (Chen et al., 2022). Volatile components in RG can inhibit contraction of the mouse uterus *in vitro* (An et al., 2009). Additionally, WZY is recommended as a primary treatment for dysmenorrhea (Hao et al., 2025). Therefore, these herbal medicines may synergistically treat PD, contributing to the therapeutic effects of WTT. WJZT is a traditional Chinese patent medicine composed of Angelica sinensis, Radix Paeoniae Alba, Rhizoma Chuanxiong, Radix Salviae Miltiorrhizae, Rhizoma Corydalis, Pollen Typhae, Evodia rutaecarpa, Cortex Cinnamomi, Asarum sieboldii, Rhizoma Cyperi, Radix Aucklandiae, and Borneol. WJZT is used clinically to warm the meridians, dispel cold, remove blood stasis, and relieve pain. Therefore, WJZT was used as the positive control to further elucidate the therapeutic effects of WTT on PD.

The pathogenesis of PD is mainly attributed to the accumulation of PGs in the uterus (Dawood, 1987). Among them, PGF_2*α*_ and PGE_2_ are the major components affecting the occurrence of PD. PGF_2*α*_ not only induces uterine contractions, which restrict blood flow, but also causes the constriction of arcuate vessels (Dawood, 2006). PGE_2_ can lead to myometrial contractions and uterine vessel constriction (Iacovides et al., 2015). In the present study, the levels of PGF_2*α*_ and PGE_2_ in the uterus of rats in the PD group increased compared with those in the CG group. Meanwhile, there was a decrease in PGF_2*α*_ and PGE_2_ levels in the WTT group, suggesting that WTT could alleviate PD by reducing the production and release of these PGs. Laser speckle contrast imaging results indicated a decrease in blood flow in the uterus, claws, and sublingual regions of rats in the PD group, whereas blood flow increased in the WTT group, which may be attributed to the decreased PGF_2*α*_ levels.

Additionally, the PD group showed increased levels of E2 and *β*-PG and decreased levels of NO, whereas WTT reversed these indicators. E2 promotes the development of secondary sexual characteristics and maturation of sexual organs. The increase in E2 levels may be attributed to the accumulated production and release of PGs (Fang et al., 2017). WTT could downregulate E2 expression by reducing PG production and release. PGs are involved in inflammatory responses (Huang et al., 2016). Therefore, WTT may inhibit inflammation by lowering PG levels. Interestingly, E2 and PG showed similar trends in content changes, which could be attributed to exogenous E2 stimulation, possibly triggering endogenous compensatory responses leading to elevated PG levels—a phenomenon previously reported (Geiger et al., 2016). Histopathological analysis also showed the presence of inflammation in the PD group, which was mitigated in the WTT group. NO is a cytokine that dilates blood vessels and supports normal blood circulation (Pan et al., 2019). WTT reversed the abnormal expression of NO, promoted blood flow, and alleviated PD. These changes in key indicators demonstrate that WTT treats PD through multiple mechanisms, including inhibition of the inflammatory response and downregulation of E2 levels.

To explore the mechanism of WTT in PD, network pharmacology and transcriptomics were used to clarify the changes in the metabolic characteristics of plasma. Network pharmacology analysis indicated that WTT mainly alleviates the inflammatory response and regulates E2 levels to treat PD by targeting signaling pathways such as the HIF-1, TNF, and IL-17 signaling pathways. These pathways are closely associated with inflammatory responses. For instance, the HIF-1 signaling pathway is a primary metabolic sensor that promotes the release of inflammatory cytokines (Tang et al., 2023). TNF is a key regulator of the inflammatory response that can trigger diverse signaling cascades and disrupt the balance and composition of signaling complexes, thereby causing inflammatory diseases (Bradley, 2008; Webster and Vucic, 2020). The IL-17 signaling pathway also is a highly versatile pro-inflammatory cytokine pathway involved in inflammatory responses (Li et al., 2019). The IL-17 signaling pathway is closely related to pain, which is the main symptom of PD (McNamee et al., 2011). IL-17 causes Ca^2+^ influx and contraction in smooth muscle cells (Akiho et al., 2013; Chesné et al., 2014). In addition, there is a potential correlation between IL-17 and PD-related indicators. For example, PGE_2_ increases the production and release of IL-17 (Napolitani et al., 2009). E2 increases the expression of the IL-17 family of cytokines (IL-17A, IL-17E, and IL-17F) (Wu et al., 2022). Interestingly, the IL-17 signaling pathway was also identified through transcriptomic analysis. Overall, the IL-17 signaling pathway was associated with PD, and was selected for further analysis. Some genes related to the IL-17 signaling pathway, such as *Cxcl6* and *Lcn2*, showed significant differences between the WTT and PD groups. Furthermore, ELISA and WB analyses confirmed changes in the levels of these indicators. Overall, WTT could downregulate the levels of Cxcl6 and IL-17, and upregulate the level of Lcn2, further regulate the IL-17 signaling pathway, ultimately alleviate the inflammatory response and treat PD.

## 5 Conclusion

This study established a rat model of PD, and implemented an intervention using WTT. Furthermore, the blood-entry components of WTT were determined using UPLC-Q-Exactive Orbitrap-MS, and the effectiveness of WTT was evaluated by assessing the writhing response, pathological changes, and levels of E2, NO, and PG. Finally, the mechanism of action of WTT in PD was elucidated using network pharmacology combined with transcriptomics. These results demonstrated that 49 original blood-entry components in WTT commonly improved PD by upregulating the levels of NO and Lcn2, and downregulating the levels of PGF_2*α*_, PGE_2_, E2, PG, Cxcl6, and IL-17, thereby inducing phenotypic changes such as the increased blood flow, mitigation of inflammatory responses, and inhibition of the writhing response. More importantly, WTT mainly downregulated the Cxcl6 and IL-17 levels, and upregulated the Lcn2 expression to alleviate inflammation, ultimately treating PD. The present study provides a reference for further research and the development of new medicines for treating PD and offers a scientific basis for the clinical application of WTT. However, the current study utilized only a single dose of WTT without dose–response evaluation. In addition, current research lacks studies on the dynamic changes in the efficacy of WTT over time. Further research should comprehensively evaluate the effects of different doses of WTT on PD and investigate the time–efficacy relation of WTT to provide a reference for individuals of different age groups.

## Data Availability

The data presented in the study are deposited in the Sequence Read Archive (SRA) in NCBI repository, accession number PRJNA1281571.
