# Supplementary material for: Interaction of blood-entry components, network pharmacology and transcriptomics to elucidate the mechanism of Wentong plaster in treating primary dysmenorrhea
Source: Front Pharmacol. 2025 Jul 2;16:1591558. doi: 10.3389/fphar.2025.1591558 (PMC12263669; doi:10.3389/fphar.2025.1591558)
Supplement: Supplementary file 1 [file DataSheet1.docx]

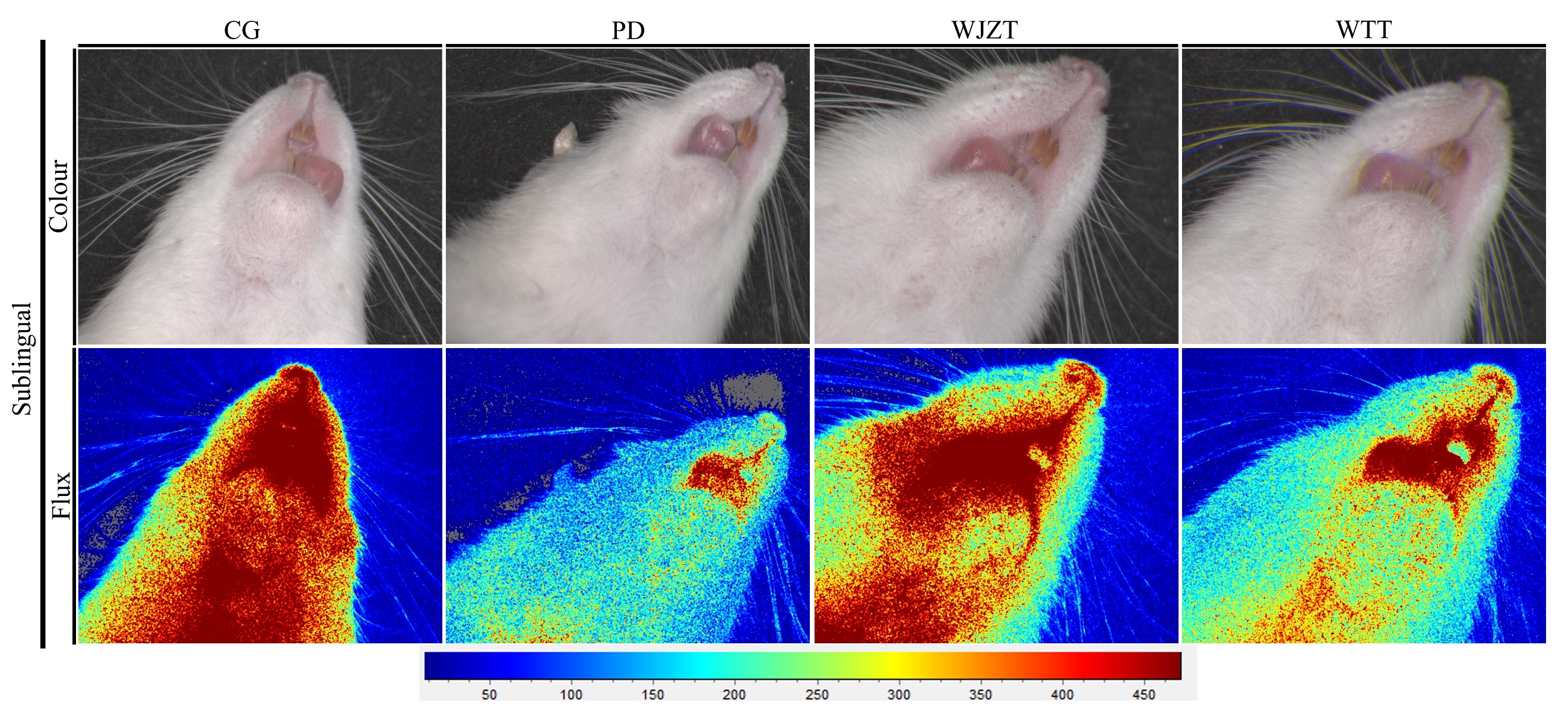


**Fig. S1** The blood flow imaging in sublingual of rats


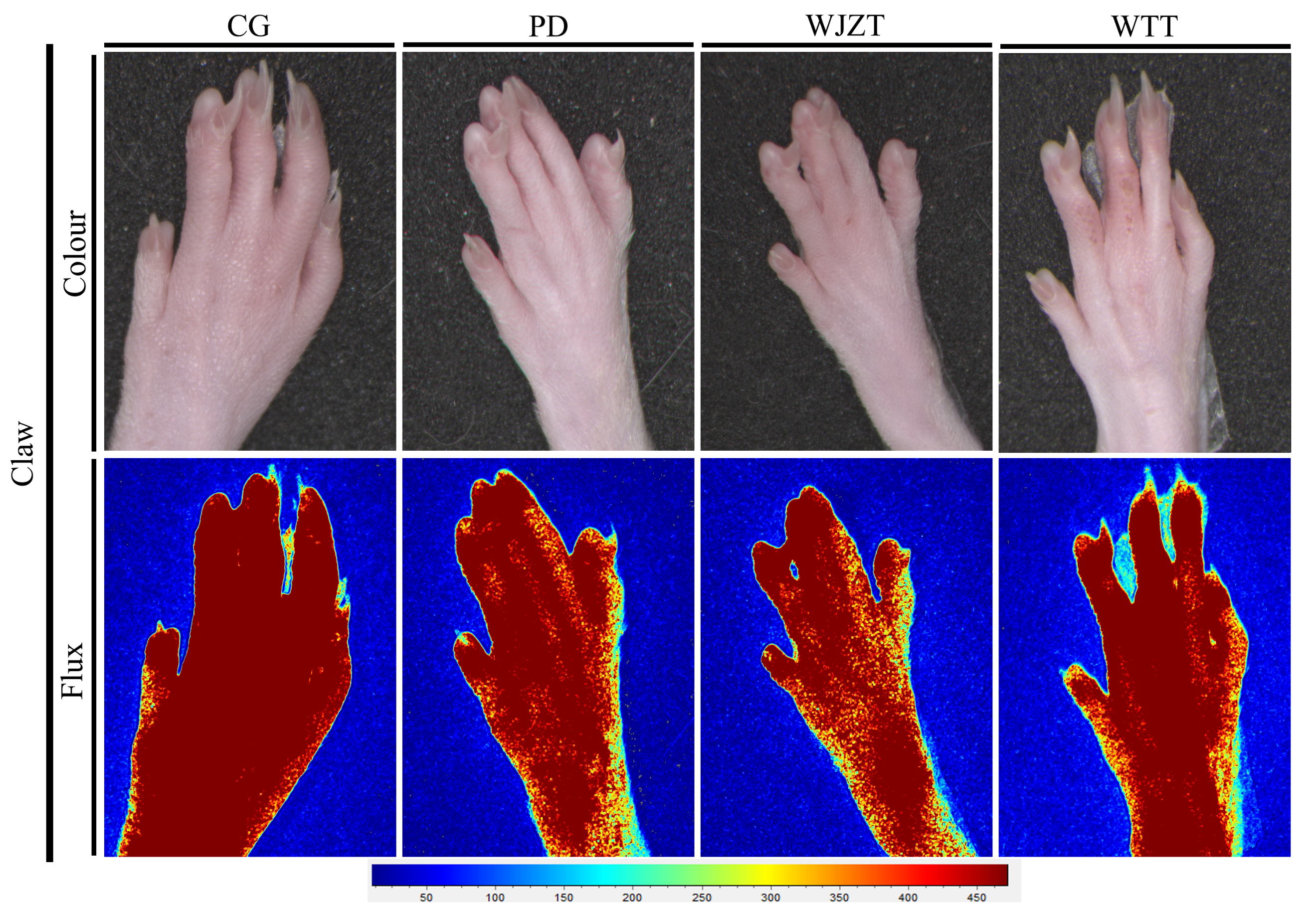


**Fig. S2** The blood flow imaging in claw of rats


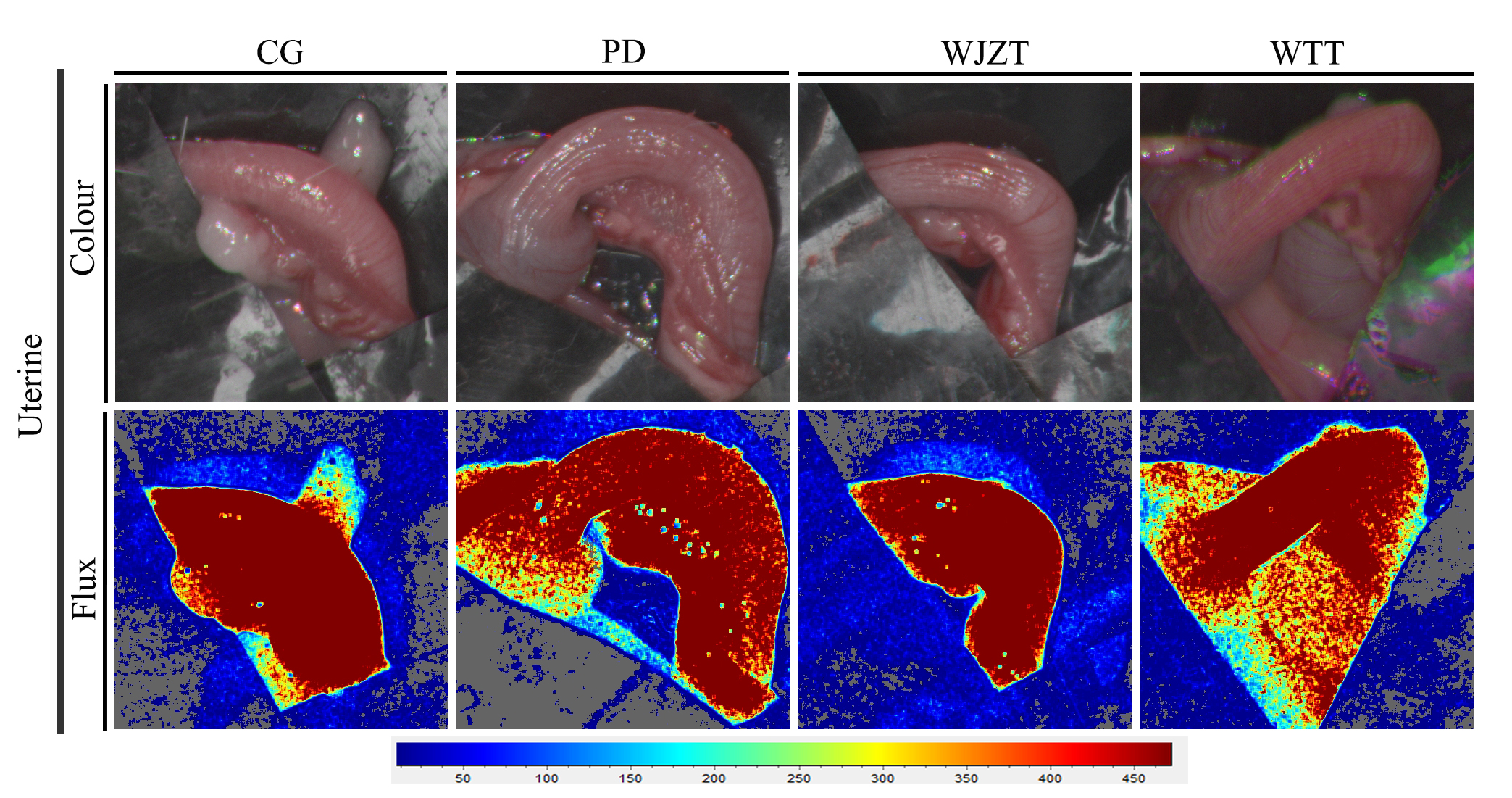


**Fig. S3** The blood flow imaging in uterine of rats

**
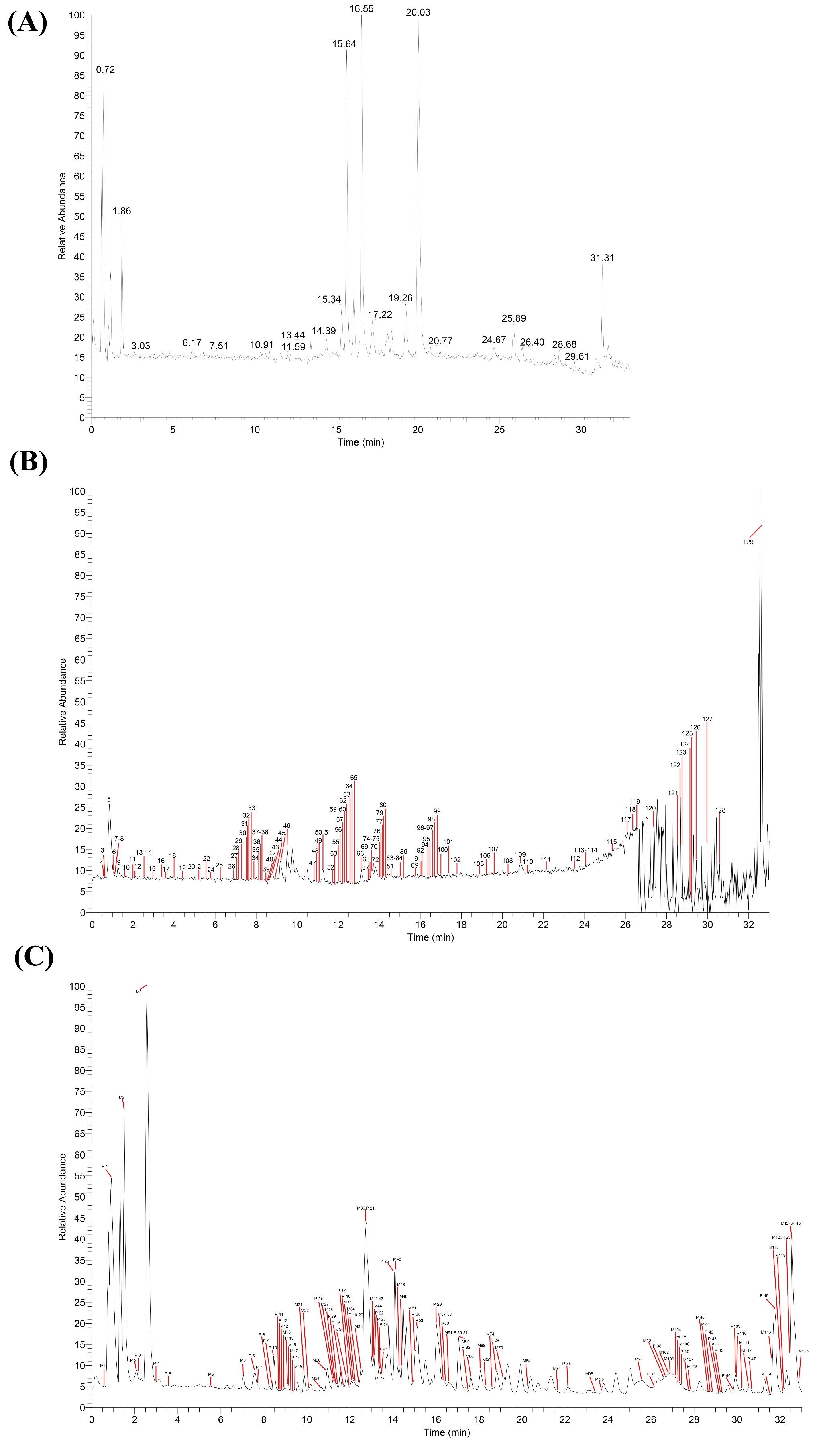
**

**Fig. S4** Total ion current diagram in positive ion mode: blank plasma (A), WTT solution (B), and plasma containing the medicine (C).

**
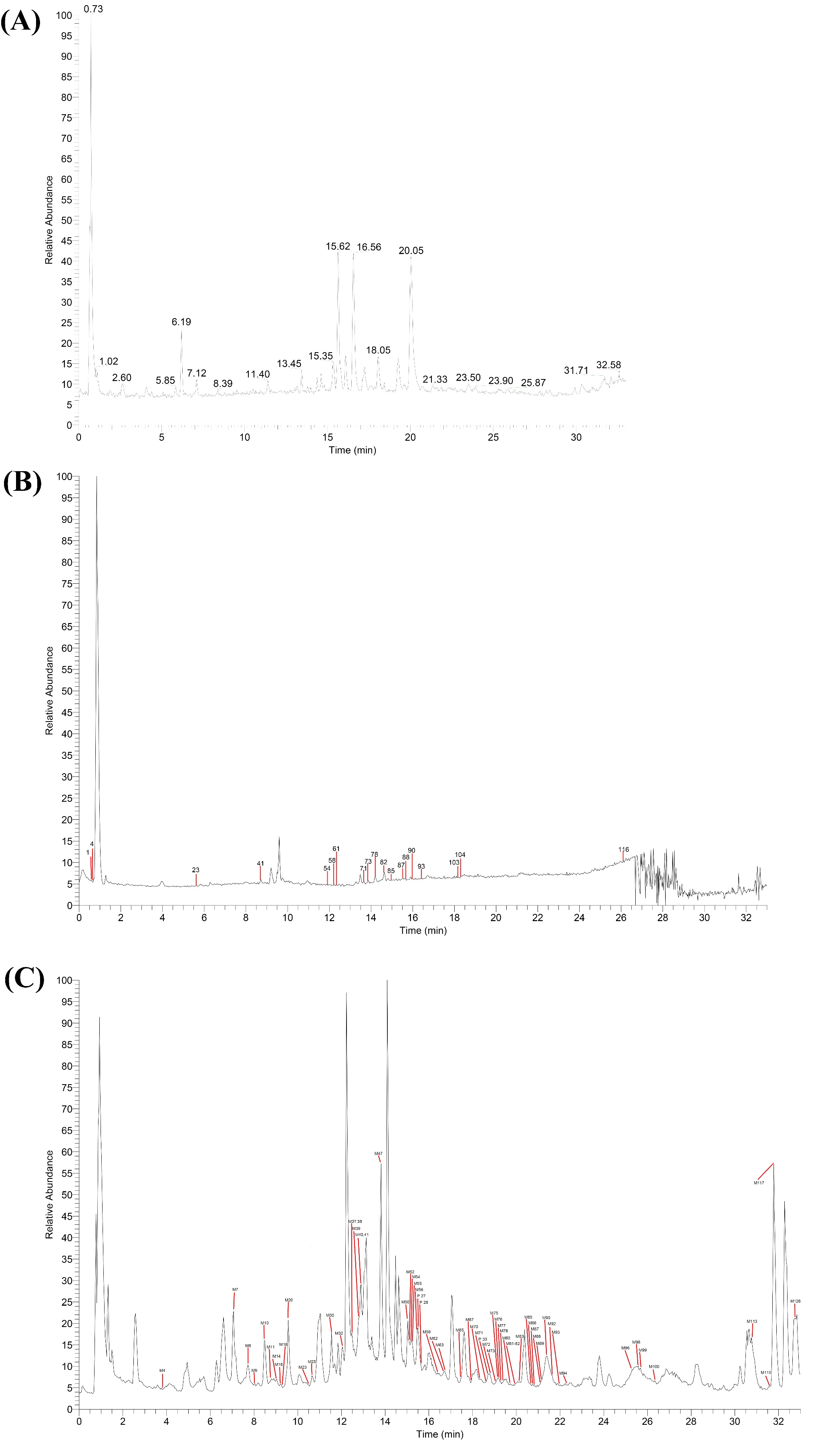
**

**Fig. S5** Total ion current diagram in negative ion mode: blank plasma (A), WTT solution (B), and plasma containing the medicine (C).


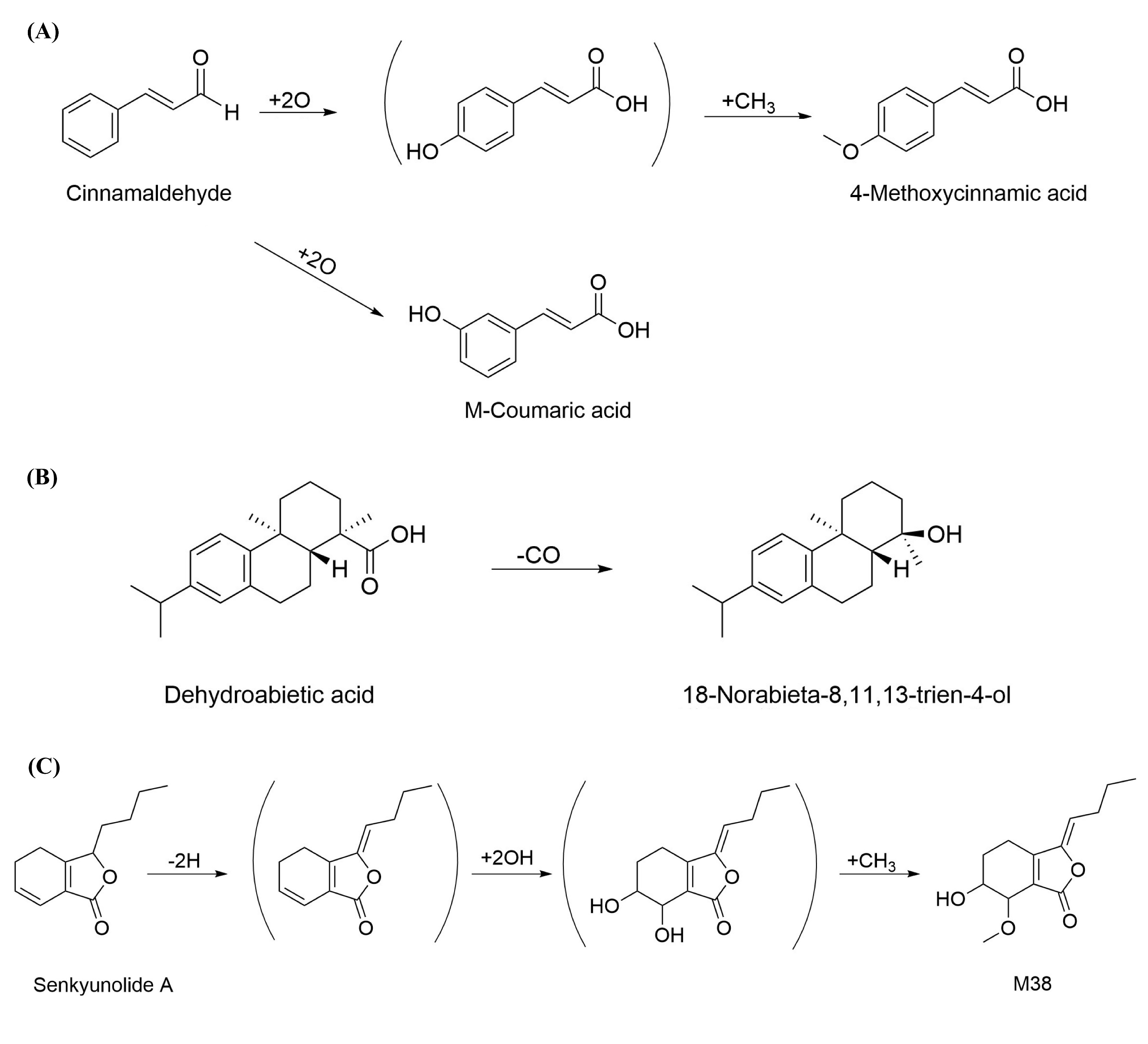


**Fig. S6** The proposed metabolic pathway: cinnamaldehyde (A); dehydroabietic acid (B); osthole A (C).


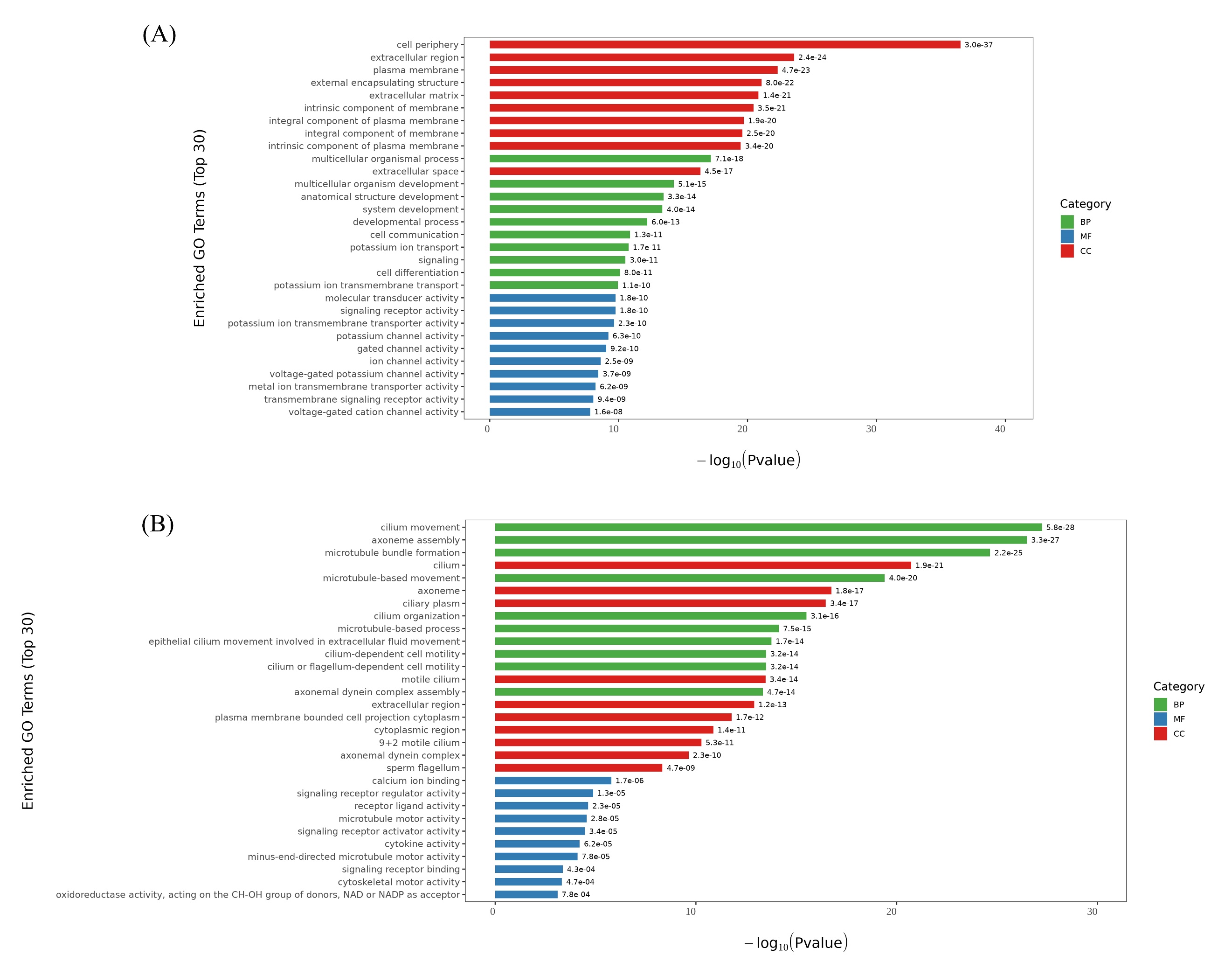


**Fig. S7** The GO enrichment analysis of DEGs between PD and CG groups (A) and WTT and PD groups (B)

**
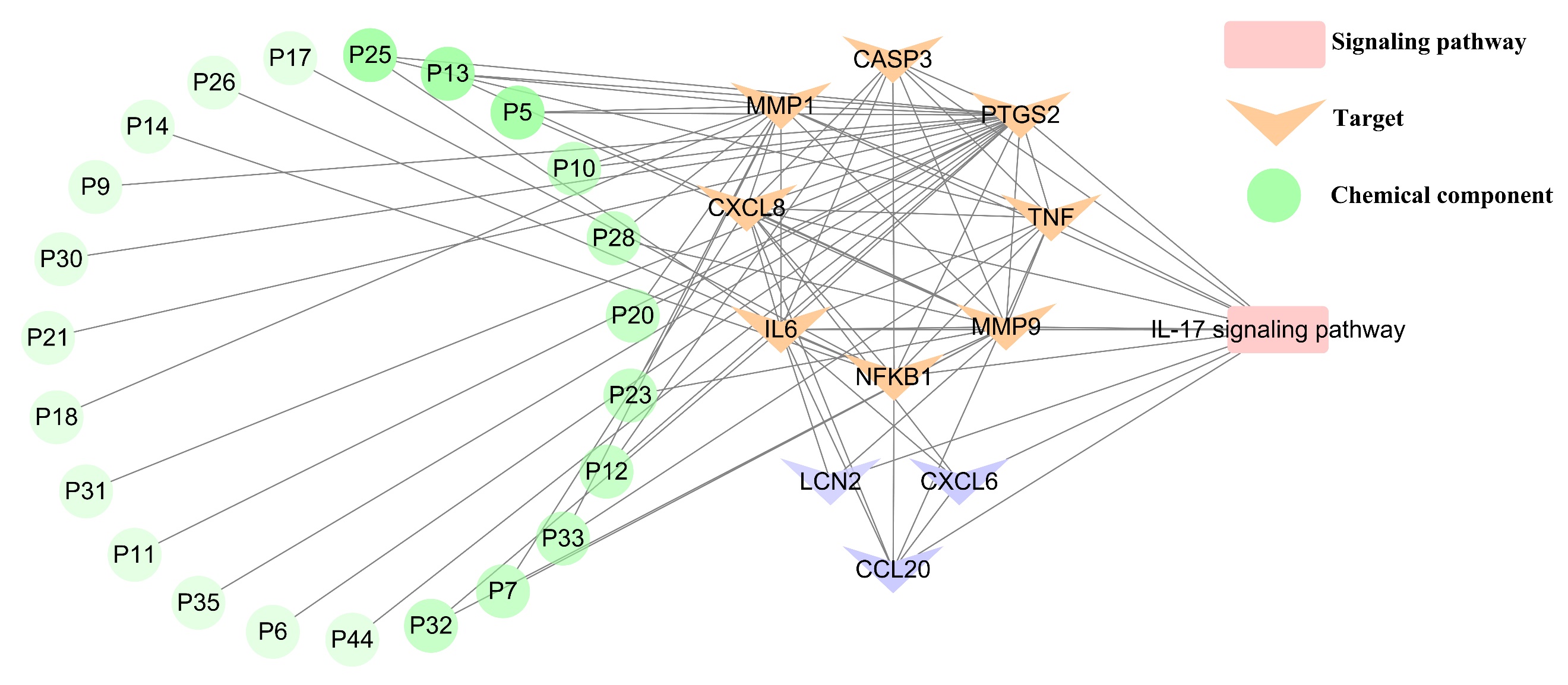
**

**Fig. S8** The “blood-entry compounds-direct/indirect targets-IL-17 signaling pathway” topological network

**Table S1** The effect of WTT on writhing response in PD rats with cold coagulation and stasis type (x ± s, n=10)

| Group | Latency (min) | Writhing frequency (n/30min) | Writhing inhibition rate (%) |
| --- | --- | --- | --- |
| CG | 30^***^ | 0^***^ | - |
| PD | 2.04±1.32 | 24.2±4.49 | - |
| WJZT | 3.42±1.59 | 15.1±5.05^**^ | 37.60 |
| WTT | 3.67±1.10^*^ | 13.9±3.45^**^ | 42.56 |

*: Compared with PD group, *: *p*<0.05, **: *p*<0.01

**Table S2** Pathological scoring of rat uterine tissue using McGuigan's criteria

| Items | Group | | | |
| --- | --- | --- | --- | --- |
|  | CG | PD | WTT | WJZT |
| Proliferation of uterine mucosal epithelial cells | 0.55±0.69 | 2.69±0.48^**^ | 1.83±0.72^##^ | 1.82±0.60^##^ |
| Hypercolumnar change in uterine mucosal epithelial cells | 0±0 | 2.44±0.63^**^ | 1.17±0.72^##^ | 1.73±0.65^#^ |
| Mucosal glandular hyperplasia | 0.64±0.50 | 2.94±0.68^**^ | 2.00±0.74^#^ | 2.18±0.60^#^ |
| Increased secretions | 0.27±0.47 | 2.69±0.79^**^ | 1.33±0.78^##^ | 1.91±0.94^#^ |
| Total score | 1.45±1.44 | 8.06±1.44^**^ | 6.33±2.42^#^ | 7.64±2.38 |

**Table S3** Detailed information of blood-entry components

| ID | Rt/min | Name | Adduct | Experimental *m/z* | Theoretical *m/z* | *δ*/ppm | Formula | Fragments |
| --- | --- | --- | --- | --- | --- | --- | --- | --- |
| M1 | 0.85 | Choline glycerophosphate | [M+K]^+^ | 296.0661 | 296.066 | 0.27 | C8H20NO6P | 190.3173, 199.1365, 296.0662, 98.8272, 67.037 |
| P1 | 1.18 | Kainic acid | [M+CH3OH+H]^+^ | 246.1337 | 246.13 | 0.34 | C10H15NO4 | 69.0342, 99.0447, 98.097, 57.0342, 67.0547 |
| M2 | 1.58 | 1-Methoxyindole-3-carboxylic acid | [M+NH4]^+^ | 209.0921 | 209.0921 | 0.33 | C10H9NO3 | 99.00813, 192.0657, 77.0394, 174.0551, 162.0545 |
| P2 | 2.22 | 1-Indanone | [M+ACN+H]^+^ | 174.0913 | 173.0835 | 0.01 | C9H8O | 69.8453, 79.0545, 88.9691, 57.0706, 67.7608 |
| P3 | 2.29 | Merucathinone | [M+ACN+H]^+^ | 217.1335 | 217.13 | -0.12 | C11H13NO | 191.3945, 68.9312, 188.1075, 174.0916, 66.2217 |
| M3 | 2.5 | Miglustat | [M+K]^+^ | 258.11 | 258.1102 | -0.89 | C10H21NO4 | 199.0371, 58.066, 98.5279, 98.9847, 181.0255 |
| P4 | 3.05 | Cinnamaldehyde | [M+ACN+H]^+^ | 174.0915 | 173.0835 | 0.86 | C9H8O | 57.0707, 57.4426, 173.9851, 174.0586, 174.0916 |
| P5 | 3.63 | Cyclo(leucylprolyl) | [M+H]^+^ | 211.144 | 211.1441 | -0.56 | C11H18N2O2 | 59.3458, 89.1783, 99.0444, 194.1172, 98.0605 |
| M4 | 3.97 | N-(1-Carboxy-2-phenylethyl)glutamine | [M-H]^-^ | 293.1144 | 293.1132 | 0.42 | C14H18N2O5 | 99.0979, 99.6795, 199.0835, 293.1182, 77.313 |
| M5 | 5.68 | 3-Hydroxycoumarin | [M+NH4]^+^ | 180.0658 | 180.0655 | 1.76 | C9H6O3 | 69.9783, 79.0549, 68.05, 98.0971, 180.0655 |
| M6 | 7.12 | 11-Beta-hydroxyandrostenedione | [M+2Na-H]^+^ | 347.1604 | 347.1594 | 3.52 | C19H26O3 | 199.1228, 297.1213, 185.1072, 185.1153, 186.1156 |
| M7 | 7.17 | Cyclo(Hyp-Val) | [M-H]^-^ | 211.1081 | 211.1077 | -3.35 | C10H16N2O3 | 67.8045, 77.6273, 162.6043, 168.1019, 55.1623 |
| P6 | 7.77 | Allocryptopine | [M+H]^+^ | 370.1647 | 370.1649 | -0.49 | C21H23NO5 | 190.0863, 191.0706, 192.102, 290.0941, 291.1014 |
| P7 | 7.8 | Lauric Acid | [M+NH4]^+^ | 218.2114 | 218.2115 | -0.46 | C12H24O2 | 69.3415, 199.6626, 58.0657, 88.0763, 98.3667 |
| M8 | 7.93 | Turkesterone | [2M+FA-H]^-^ | 1037.601 | 1037.604 | -4.14 | C27H44O8 | 198.8627, 199.6014, 298.2482, 993.5754, 996.5067 |
| M9 | 8 | Oroxylin A-7-O-Beta-D-glucuronide | [M-H]^-^ | 459.0937 | 459.0922 | 0.98 | C22H20O11 | 59.0123, 69.7738, 89.0234, 99.0076, 394.6461 |
| P8 | 8.31 | 12-Hydroxydodecanoic acid | [M+NH4]^+^ | 234.2063 | 234.2064 | -0.42 | C12H24O3 | 69.0709, 58.0659, 88.0713, 88.0764, 186.185 |
| P9 | 8.56 | Tetrahydroberberine THB | [M+H]^+^ | 340.1543 | 340.1543 | -0.21 | C20H21O4N | 79.0548, 190.0868, 292.1024, 294.0887, 294.1171 |
| M10 | 8.58 | 3,4-Dimethoxybenzenepropanamine | [M+FA-H]^-^ | 240.1238 | C43H58N3O8 | -1.83 | C11H17NO2 | 69.0333, 69.2346, 196.1335, 196.1842, 199.2545 |
| P10 | 8.6 | 3alpha-Hydroxytanshinone IIA | [M+ACN+H]^+^ | 352.1543 | 352.1543 | -0.26 | C19H18O4 | 59.053, 69.2622, 194.5473, 291.0892, 291.1259 |
| M11 | 8.72 | Genistin | [M-H]^-^ | 431.0986 | 431.0973 | 0.56 | C21H20O10 | 59.0125, 89.0231, 99.0074, 185.7572, 57.033 |
| P11 | 8.78 | Corydaline | [M+H]^+^ | 370.2011 | 370.2013 | -0.37 | C22H27NO4 | 79.0548, 190.0863, 191.0956, 192.1021, 193.9188 |
| P12 | 8.89 | Karanjin | [M+ACN+H]^-^ | 334.1071 | 334.9021 | -0.87 | C18H12O4 | 199.4312, 290.0818, 291.0894, 289.0742, 276.1023 |
| M12 | 8.93 | M-Coumaric acid | [M+ACN+H]^+^ | 206.0815 | 206.0812 | 2.28 | C9H8O3 | 58.4665, 68.0155, 88.0218, 188.0707, 170.0603 |
| M13 | 9.03 | Apigenin 7-O-glucuronide | [M+H]^+^ | 447.093 | 447.0922 | 1.87 | C21H18O11 | 197.0602, 68.9977, 185.0589, 187.0386, 187.0752 |
| M14 | 9.05 | Genistein | [M-H]^-^ | 269.0454 | 269.0444 | -0.53 | C15H10O5 | 196.053, 197.0599, 180.0578, 181.0646, 183.0438 |
| P13 | 9.11 | Cis-N-Feruloyltyramine | [M+H]^+^ | 314.1384 | 314.1387 | -0.82 | C18H19NO4 | 69.1685, 89.8118, 57.286, 177.0548, 178.9181 |
| M15 | 9.18 | 6,7-Dihydroneridienone A | [M+NH4]^+^ | 346.2382 | 346.2377 | 1.51 | C21H28O3 | 195.1175, 197.133, 293.1891, 299.2001, 68.0502 |
| M16 | 9.34 | Megastigm-7-ene-3,4,6,9-tetrol | [M+FA-H]^-^ | 289.1658 | 289.1646 | 0.75 | C13H24O4 | 199.158, 68.8024, 98.4998, 181.3957, 289.1661 |
| M17 | 9.36 | Hexyl glucoside | [M+H-2H2O]^+^ | 229.1438 | 229.1434 | 1.28 | C12H24O6 | 69.0706, 79.1671, 99.081, 78.2438, 88.0764 |
| P14 | 9.4 | 3-Hydroxymethylenetanshinquinone | [M+ACN+H]^+^ | 336.1227 | 336.123 | -1.1 | C18H14O4 | 194.6088, 199.6125, 291.0881, 292.097, 88.0484 |
| M18 | 9.42 | Nonanoic acid | [M+FA-H]^-^ | 203.1282 | 203.1278 | -4.26 | C9H18O2 | 69.0333, 99.0801, 185.1174, 57.0331, 87.044 |
| M19 | 9.58 | Hydrangenol 8-O-glucoside | [M+H-2H2O]^+^ | 436.161 | 436.1602 | 1.84 | C21H22O9 | 59.0498, 199.0394, 181.0649, 187.0393, 188.047 |
| M20 | 9.67 | Alepterolic acid | [M+FA-H]^-^ | 365.2332 | 365.2323 | -0.46 | C20H32O3 | 59.3465, 193.0819, 57.4792, 273.1856, 275.2016 |
| M21 | 9.98 | Thymic acid | [M+NH4]^+^ | 168.1386 | 168.1383 | 2.04 | C10H14O | 69.0341, 69.0706, 79.0549, 98.0607, 98.0964 |
| M22 | 10.19 | Antibiotic AB 4015B | [M+2Na-H]^+^ | 458.2654 | 458.2642 | 2.9 | C26H39NO3 | 69.0454, 196.0728, 199.1113, 298.2166, 183.1166 |
| M23 | 10.54 | Sarcostin | [M-H]^-^ | 381.2285 | 381.2272 | 0.58 | C21H34O6 | 58.2327, 181.1224, 381.2284, 76.6978, 163.1112 |
| M24 | 10.67 | 2,4,4'-Trihydroxydihydrochalcone | [M+H]^+^ | 259.0969 | 259.0965 | 1.66 | C15H14O4 | 79.055, 195.08, 198.0664, 68.9978, 88.5239 |
| M25 | 10.69 | Purpuride | [M+Cl]^-^ | 426.2037 | 426.2042 | -4.06 | C22H33NO5 | 69.8642, 382.2136, 85.5192, 95.4042, 63.0869 |
| M26 | 11.06 | Estrone sulfate | [M+2Na-H]^+^ | 315.1341 | 315.1331 | 3.5 | C18H22O2 | 59.1197, 198.1145, 199.0864, 199.1229, 297.1243 |
| P15 | 11.24 | 1-Hexanol | [2M+ACN+H]^+^ | 246.2428 | 246.2428 | 0.34 | C6H14O | 199.5702, 68.05, 88.0763, 88.0822, 184.2064 |
| M27 | 11.32 | Cryptomoscatone D2 | [M+CH3OH+H]^+^ | 321.17 | 321.1697 | 1.06 | C17H20O4 | 69.0343, 99.0446, 57.0707, 97.0288, 97.0764 |
| M28 | 11.42 | Antcin A | [M+ACN+Na]^+^ | 518.3233 | 518.3241 | -1.67 | C29H42O4 | 79.0549, 196.0725, 199.1495, 184.072, 185.1329 |
| M29 | 11.57 | 3-Indolepropionic acid | [M+H]^+^ | 189.0788 | 189.0784 | 2.05 | C11H10NO2 | 69.593, 188.0725, 189.0786, 77.0392, 171.0679 |
| M30 | 11.69 | Lucidenic acid SP1 | [M-H]^-^ | 459.2755 | 459.2741 | 0.52 | C27H40O6 | 69.7807, 397.274, 459.2753, 53.39, 132.6813 |
| P16 | 11.85 | Oxoglaucine | [M+H]^+^ | 352.1176 | 352.1179 | -0.9 | C20H17NO5 | 190.5258, 292.0612, 292.9998, 293.0685, 294.0759 |
| M31 | 11.93 | 4-Methoxycinnamic acid | [M+H-H2O]^+^ | 161.0599 | 161.0597 | 1.1 | C10H10O3 | 59.0939, 69.0706, 79.0549, 89.0393, 78.9312 |
| P17 | 12.07 | Macrophylline | [M+H-2H2O]^+^ | 204.1384 | 204.1383 | 0.28 | C13H21NO3 | 59.3668, 69.0706, 69.442, 79.0548, 199.5761 |
| P18 | 12.14 | Hainanolidol | [M+H]^+^ | 313.143 | 313.1434 | -1.28 | C19H20O4 | 59.0499, 69.6186, 191.0853, 192.0942, 193.1013 |
| M32 | 12.16 | 13-Methyl-8,11,13-podocarpatriene-3,12-diol | [M+FA-H]^-^ | 319.1913 | 319.1904 | -0.85 | C18H26O2 | 69.0326, 69.9811, 89.1048, 294.7765, 78.9576 |
| M33 | 12.32 | 4,5-Dihydroblumenol A | [M+NH4]^+^ | 244.1911 | 244.1907 | 1.72 | C13H22O3 | 69.0706, 79.0544, 198.1676, 198.1854, 199.2884 |
| M34 | 12.39 | Dextrothyroxine | [M+H]^+^ | 777.6955 | 777.694 | 2.02 | C15H11I4NO4 | 69.6859, 196.0535, 196.076, 197.06, 181.0653 |
| P19 | 12.6 | Obscuraminol C | [M+NH4]^+^ | 271.2742 | 271.2744 | -0.62 | C16H31NO | 89.1079, 183.1748, 57.0707, 67.055, 271.1304 |
| P20 | 12.6 | Ajugalide D | [M+H-2H2O]^+^ | 345.2065 | 345.206 | 1.28 | C21H32O6 | 69.0706, 79.0549, 195.1175, 197.1331, 291.1754 |
| M35 | 12.64 | 3-Pyridineacetic acid | [M+H-H2O]^+^ | 120.0447 | 120.0444 | 2.14 | C7H7NO2 | 78.9313, 56.0501, 96.9415, 55.055, 65.0393 |
| M36 | 12.81 | Undecylenic acid | [M+H]^+^ | 185.1537 | 185.1536 | 0.29 | C11H20O2 | 59.0499, 69.0343, 69.0706, 69.8573, 99.0811 |
| P21 | 12.81 | Parthenolide | [M+H-H2O]^+^ | 249.1483 | 231.138 | -0.76 | C15H20O3 | 69.0342, 69.0707, 79.0549, 185.1328, 189.1277 |
| M37 | 12.9 | 17beta-Estradiol 3-sulfate | [M+FA-H]^-^ | 317.1756 | 317.1747 | -1.02 | C18H24O2 | 59.0329, 69.033, 69.9849, 193.0062, 193.086 |
| M38 | 12.9 | 6-Hydroxy-7-methoxydihydroligustilide | [M+FA-H]^-^ | 283.1186 | 283.1176 | -0.5 | C13H18O4 | 59.0124, 59.6935, 69.5801, 191.1071, 194.9886 |
| M39 | 12.93 | 3,7-O-Diacetylpinobanksin | [M+FA-H]^-^ | 401.0876 | 401.0867 | -0.57 | C19H16O7 | 69.9471, 292.2248, 58.1461, 180.0569, 181.0649 |
| M40 | 12.97 | alpha-Desmotroposantonin | [M+FA-H]^-^ | 291.1238 | 291.1227 | 0.03 | C15H18O3 | 59.9027, 69.8115, 198.9963, 199.2851, 291.0038 |
| M41 | 12.97 | Chaetoglobosin A | [M+Na-2H]^-^ | 548.2317 | 548.2282 | 4.55 | C32H35N2O5 | 69.6053, 99.9245, 199.5379, 58.3852, 484.2141 |
| M42 | 13.05 | Taurochenodeoxycholic Acid | [M+H-H2O]^+^ | 482.2937 | 482.2935 | 0.45 | C26H45NO6S | 69.0705, 69.7223, 79.055, 199.1486, 185.1332 |
| M43 | 13.05 | Teucrin A | [M+H-2H2O]^+^ | 309.1122 | 309.1121 | 0.26 | C19H20O6 | 191.0855, 192.093, 193.1009, 194.1094, 195.0809 |
| M44 | 13.09 | Altamisic acid | [M+H-2H2O]^+^ | 245.1174 | 245.1172 | 0.52 | C15H20O5 | 59.0499, 69.0343, 79.0549, 181.1014, 182.1093 |
| P22 | 13.12 | Ursocholic acid | [M+H-2H2O]^+^ | 373.2738 | 373.2737 | 0.25 | C24H40O5 | 69.0706, 79.0549, 99.0446, 193.1228, 195.1376 |
| P23 | 13.55 | Bisabolangelone | [M+H-H2O]^+^ | 231.1381 | 249.1485 | 0.72 | C15H20O3 | 69.0707, 79.055, 185.0972, 185.1328, 189.0915 |
| P24 | 13.59 | Uzarigenin | [M+H-2H2O]^+^ | 339.2317 | 339.232 | -0.48 | C23H34O4 | 69.0339, 69.8527, 283.1696, 57.0707, 97.0134 |
| M45 | 13.69 | 1-Octanol | [2M+ACN+H]^+^ | 302.3052 | 302.3054 | -0.55 | C8H18O | 69.0705, 69.8124, 79.7724, 199.3016, 68.0503 |
| P25 | 14.11 | 3-hydroxy-12-oxochol-9(11)-en-24-oic acid | [M+H]^+^ | 389.2684 | 389.269 | -0.48 | C24H36O4 | 69.0706, 79.055, 195.1379, 197.1327, 199.1481 |
| M46 | 14.14 | 3-Oxocholic acid | [M+H-2H2O]^+^ | 371.2582 | 371.2581 | 0.4 | C24H38O5 | 79.0539, 99.0444, 193.1225, 197.1326, 199.1481 |
| M47 | 14.26 | 3beta,5alpha,9alpha-Trihydroxyergosta-7,22-dien-6-one | [M+FA-H]^-^ | 489.3222 | 489.3211 | -0.02 | C28H44O4 | 59.0123, 89.7965, 292.6101, 282.7585, 283.1098 |
| M48 | 14.32 | Nonadecanoic acid | [M+NH4]^+^ | 316.321 | 316.321 | 0.1 | C19H38O2 | 59.4279, 69.8182, 298.31, 88.0763, 98.2354 |
| M49 | 14.49 | Chenodeoxycholic acid | [M+H-H2O]^+^ | 375.2894 | 391.284 | 0.07 | C24H40O4 | 69.0707, 79.0547, 195.1382, 68.6279, 185.1326 |
| M50 | 15 | Coronarin A | [M+FA-H]^-^ | 345.2071 | 345.206 | -0.06 | C20H28O2 | 69.7502, 79.4742, 190.9909, 196.174, 199.3502 |
| M51 | 15.09 | 4-Hydroxy-11,12,13-trinor-5-eudesmen-7-one | [2M+H]^+^ | 389.2686 | 239.1278 | -0.12 | C12H18O2 | 69.0704, 79.055, 183.1172, 185.1327, 189.1274 |
| M52 | 15.15 | Poststerone | [M+Na-2H]^-^ | 383.1841 | 383.1829 | 0.29 | C21H30O5 | 69.906, 190.9915, 383.1836, 67.9685, 97.4021 |
| P26 | 15.16 | Lathyrol | [M+H-2H2O]^+^ | 299.2004 | 299.201 | -0.54 | C20H30O4 | 69.0706, 69.9885, 79.0549, 193.1227, 195.1178 |
| M53 | 15.2 | Dehydroandrographolide | [M+H-2H2O]^+^ | 297.1852 | 297.1849 | 0.84 | C20H28O4 | 69.0706, 79.055, 193.1015, 194.11, 195.1171 |
| M54 | 15.22 | Taurodeoxycholate sodium salt | [M+Cl]^-^ | 566.2768 | 556.247 | -0.29 | C26H44NNaO6S | 69.5784, 79.5732, 79.956, 498.2893, 498.3665 |
| M55 | 15.29 | Yunnancoronarin A | [M+FA-H]^-^ | 345.2071 | 345.206 | -0.19 | C20H28O2 | 59.0259, 89.1655, 99.9244, 191.5984, 78.9578 |
| M56 | 15.5 | Convallagenin B | [M-H2O-H]^-^ | 445.2958 | 445.2949 | -0.25 | C27H44O6 | 69.8187, 197.8793, 198.5973, 199.6221, 293.2119 |
| P27 | 15.53 | Desoxycholic acid | [M-H]^-^ | 391.2855 | 391.2843 | 0.23 | C24H40O4 | 69.0332, 89.6505, 391.2852, 68.9155, 286.9951 |
| P28 | 15.61 | 8(14),15-Isopimaradiene-3,18-diol | [M+FA-H]^-^ | 349.2385 | 349.2373 | 0.12 | C20H32O2 | 59.0122, 69.1323, 69.2445, 68.4589, 78.9576 |
| P29 | 16.19 | Ambroxide | [M+NH4]^+^ | 254.2479 | 254.248 | 0.46 | C16H28O | 69.0706, 79.0549, 99.0808, 198.1858, 184.1702 |
| M57 | 16.34 | Kahweol | [M+CH3OH+H]^+^ | 347.2219 | 347.2217 | 0.65 | C20H26O3 | 79.0548, 195.1168, 197.132, 293.1897, 183.1172 |
| M58 | 16.34 | 3-Amino-3-(hydroxymethyl)-1-(4-octylphenyl)-1,4-butanediol | [M+Na]^+^ | 324.2535 | 324.2533 | 0.42 | C19H33NO3 | 68.528, 88.076, 181.2169, 185.2223, 87.2679 |
| M59 | 16.43 | Andrographolide | [M-H]^-^ | 349.2022 | 349.201 | 0.37 | C20H30O5 | 191.1327, 198.9681, 293.466, 287.2013, 288.9897 |
| M60 | 16.45 | 4-Hydroxysapriparaquinone | [M+H]^+^ | 331.1906 | 331.1904 | 0.71 | C20H26O4 | 69.0706, 79.0547, 191.0705, 187.0756, 285.1849 |
| M61 | 16.63 | 18-Norabieta-8,11,13-trien-4-ol | [M+H-H2O]^+^ | 255.211 | 255.2107 | 0.97 | C19H28O | 69.0706, 79.0548, 197.1326, 199.1484, 183.1173 |
| P30 | 16.63 | Paullinic acid | [M+NH4]^+^ | 328.3208 | 328.321 | -0.76 | C20H38O2 | 69.0706, 79.0547, 199.2286, 199.5706, 199.7833 |
| P31 | 16.63 | Dehydroabietic acid | [M+H-H2O]^+^ | 301.2164 | 301.216 | 0.64 | C20H28O2 | 69.0706, 79.0549, 193.1222, 197.1329, 199.1484 |
| M62 | 16.72 | Clareolide | [M+FA-H]^-^ | 295.1915 | 295.1904 | -0.11 | C16H26O2 | 59.0124, 191.5162, 193.6276, 199.2825, 199.3449 |
| M63 | 16.86 | Didecyl phthalate | [M+FA-H]^-^ | 491.3381 | 491.3367 | 0.62 | C28H46O4 | 69.812, 89.3704, 392.225, 491.2785, 491.3375 |
| M64 | 17.13 | Methyl Linolenate | [M+H-2H2O]^+^ | 257.2265 | 257.2264 | 0.43 | C19H32O2 | 69.0706, 79.055, 197.1337, 199.1491, 185.1323 |
| P32 | 17.53 | Tenacigenin B | [M+H]^+^ | 347.222 | 347.222 | 0.8 | C21H32O5 | 69.0706, 79.055, 199.1121, 297.1851, 185.0956 |
| M65 | 17.58 | Ent-Kauran-17,19-dioic acid | [M-H]^-^ | 333.2071 | 333.206 | -0.02 | C20H30O4 | 58.4962, 57.8462, 67.1714, 67.9482, 161.4415 |
| M66 | 17.78 | Abietic Acid | [M+H]^+^ | 303.232 | 303.2319 | 0.55 | C20H30O2 | 69.0706, 69.8382, 79.0549, 193.1224, 199.1485 |
| M67 | 18.05 | 17-methylandrostane-3,6,17-triol | [M+FA-H]^-^ | 367.2489 | 367.2479 | -0.16 | C20H34O3 | 59.0123, 69.0331, 69.6185, 97.0281, 97.088 |
| M68 | 18.18 | 3alpha-Akebonoic Acid | [M+H-H2O]^+^ | 441.3367 | 441.3363 | 0.78 | C29H44O3 | 69.0706, 79.055, 89.5813, 191.1795, 395.3318 |
| M69 | 18.33 | Eurycomalide A | [M+H-2H2O]^+^ | 315.1593 | 315.1591 | 0.48 | C19H26O6 | 69.0705, 191.0858, 193.1009, 195.0807, 197.0961 |
| M70 | 18.45 | Cryptomeridiol 11-rhamnoside | [M-H2O-H]^-^ | 367.2489 | 367.2479 | -0.35 | C21H38O6 | 59.0124, 69.0331, 78.9577, 189.1629, 97.089 |
| M71 | 18.64 | 7-Ketoisodrimenin | [M+Cl]^-^ | 283.1105 | 283.1095 | -0.49 | C15H20O3 | 59.3563, 69.6054, 199.0483, 199.1274, 199.3362 |
| P33 | 18.75 | Lancifodilactone F | [M+Na-2H]^-^ | 457.2572 | 457.2561 | 0.08 | C25H40O6 | 69.7868, 183.1682, 287.5122, 389.27, 77.3584 |
| M72 | 19 | Aphidicolin | [M-H]^-^ | 337.2386 | 337.2373 | 0.53 | C20H34O4 | 59.0122, 59.4387, 69.9207, 180.4978, 97.0283 |
| M73 | 19.18 | 3-(tetrahydro-2H-pyran-2-yloxy)pregnan-21-al | [M+FA-H]^-^ | 447.3118 | 447.3105 | 0.47 | C26H42O3 | 89.7488, 99.4344, 198.9446, 199.0074, 68.1399 |
| M74 | 19.23 | 17alpha-Hydroxypregnenolone | [M+H-H2O]^+^ | 315.232 | 315.2319 | 0.33 | C21H32O3 | 69.0705, 79.0548, 191.1425, 193.2139, 197.1328 |
| M75 | 19.25 | Cabraleahydroxylactone acetate | [M-H2O-H]^-^ | 455.317 | 455.3156 | 0.61 | C29H46O5 | 69.9222, 79.0782, 391.3011, 393.2801, 395.2958 |
| M76 | 19.29 | 3-oxo-21-alpha-methoxy-24-25-26-27-tetranortirucall-7-ene-23-21-lactone | [M-H]^-^ | 427.2852 | 427.2843 | -0.43 | C27H40O4 | 69.3113, 69.8665, 199.2764, 292.2731, 281.2488 |
| M77 | 19.39 | Avocadyne | [M-H2O-H]^-^ | 265.2173 | 297.206 | 0.14 | C17H32O3 | 69.7408, 58.995, 96.9586, 265.1484, 265.2179 |
| P34 | 19.45 | Nitogenin | [M+H-H2O]^+^ | 397.3102 | 397.3101 | 0.24 | C27H42O3 | 69.0706, 79.0549, 199.1483, 397.3101, 183.1174 |
| M78 | 19.47 | Polyporusterone A | [M-H]^-^ | 477.322 | 477.3211 | -0.36 | C28H46O6 | 69.4101, 199.2673, 58.0176, 78.1862, 86.0237 |
| M79 | 19.67 | Deacetylxylopic acid | [M+H-H2O]^+^ | 301.2164 | 301.2162 | 0.56 | C20H30O3 | 69.0705, 79.0547, 195.1169, 199.1111, 181.1012 |
| M80 | 19.84 | 1-O-Acetyl britannilactone | [M-H2O-H]^-^ | 289.1446 | 289.1434 | 0.26 | C17H24O5 | 289.1448, 57.0892, 87.0436, 87.1379, 175.1119 |
| M81 | 20.05 | Androstenediol | [M+FA-H]^-^ | 335.2227 | 335.2217 | -0.28 | C19H30O2 | 59.0125, 59.0904, 69.0336, 69.6051, 69.9669 |
| M82 | 20.05 | Garcinoic acid | [M-H]^-^ | 425.2698 | 425.2686 | 0.21 | C27H38O4 | 69.873, 193.2857, 381.28, 97.0795, 161.118 |
| M83 | 20.31 | 9,11-Dehydroergosterol peroxide | [M+FA-H]^-^ | 471.3114 | 471.3105 | -0.57 | C28H42O3 | 196.1948, 397.3105, 184.5582, 288.9088, 67.9424 |
| M84 | 20.36 | Yucalexin P-17 | [M+H-H2O]^+^ | 301.2161 | 301.2162 | -0.32 | C20H30O3 | 69.0705, 79.0547, 197.1326, 199.1489, 181.1016 |
| M85 | 20.7 | Vitexilactone | [M-H2O-H]^-^ | 359.2229 | 359.2217 | 0.23 | C22H34O5 | 59.0125, 69.0331, 69.6706, 297.2207, 57.9066 |
| M86 | 20.74 | Ent-3beta-Hydroxykaur-16-en-19-oic acid | [M-H]^-^ | 317.2121 | 317.2111 | -0.34 | C20H30O3 | 59.0124, 59.6265, 89.2837, 299.2001, 97.98 |
| M87 | 20.85 | 6-O-Ethyltetradymodiol | [M+K-2H]^-^ | 315.1368 | 315.1357 | 0.07 | C17H26O3 | 59.3993, 69.5963, 89.5925, 193.776, 180.0351 |
| M88 | 21.25 | Ruscogenin | [M-H]^-^ | 429.3009 | 429.2999 | -0.24 | C27H42O4 | 69.488, 69.8614, 197.0838, 199.1654, 199.2115 |
| M89 | 21.29 | 7,15-Dihydroxypodocarp-8(14)-en-13-one | [M-H]^-^ | 277.1808 | 277.1798 | -0.29 | C17H26O3 | 59.6379, 199.2509, 57.0544, 97.0876, 177.0918 |
| M90 | 21.57 | 3-Acetoxy-8(17),13E-labdadien-15-oic acid | [M-H]^-^ | 361.2384 | 361.2373 | 2.95 | C22H34O4 | 59.0124, 58.9679, 68.7417, 78.0378, 77.8504 |
| M91 | 21.63 | Micranoic acid A | [M+H-H2O]^+^ | 327.2321 | 327.2319 | 0.59 | C22H32O3 | 69.0705, 79.0547, 89.06, 199.1493, 199.166 |
| M92 | 21.64 | Delta-Tocotrienol | [M+FA-H]^-^ | 441.301 | 441.2999 | -0.1 | C27H40O2 | 69.8314, 79.6195, 397.3111, 97.0808, 441.3002 |
| M93 | 21.9 | Yibeinoside A | [M-H2O-H]^-^ | 556.362 | 556.3633 | -4.02 | C33H53NO7 | 69.5912, 199.3946, 496.342, 78.9576, 56.3379 |
| P35 | 22.14 | Cis-10-Heptadecenoic acid | [M+H-2H2O]^+^ | 233.2265 | 233.226 | 0.59 | C17H32O2 | 69.0705, 79.055, 191.1798, 57.0708, 67.055 |
| M94 | 22.45 | 15,16-Epoxy-15-ethoxy-6beta,13-dihydroxylabd-8-en-7-one | [M-H]^-^ | 379.2489 | 379.2479 | -0.23 | C22H36O5 | 59.0124, 69.7711, 99.0804, 99.5918, 58.0705 |
| M95 | 23.46 | Juvabione | [M+H-H2O]^+^ | 326.2692 | 326.269 | 0.75 | C19H32O3 | 69.0709, 79.0549, 291.1942, 294.2429, 58.9223 |
| P36 | 23.64 | (Z)-1-Methyl-2-(undec-6-enyl)quinolin-4(1H)-one | [M+H]^+^ | 312.2319 | 312.232 | -0.96 | C21H29NO | 69.6339, 297.8933, 186.0916, 187.0991, 284.7221 |
| M96 | 25.33 | Grevillol | [M+FA-H]^-^ | 337.2383 | 337.2373 | -0.41 | C19H32O2 | 293.249, 78.5753, 179.8973, 275.2386, 56.8478 |
| M97 | 25.56 | (15:2)-Anacardic acid | [M+H-H2O]^+^ | 327.2319 | 327.2319 | 0.13 | C22H32O3 | 69.0704, 79.055, 197.1326, 199.1491, 291.2114 |
| M98 | 25.69 | 14-O-Acetylsachaconitine | [M-H2O-H]^-^ | 414.2648 | 414.2639 | -0.44 | C25H39NO5 | 69.5784, 79.7057, 384.2531, 77.0705, 361.7793 |
| M99 | 25.8 | Lysofungin | [M-H]^-^ | 595.2893 | 595.2878 | 0.71 | C27H49O12P | 69.5378, 69.545, 198.6652, 595.2911, 78.9576 |
| P37 | 26.29 | Halaminol A | [M+H]^+^ | 228.2323 | 228.232 | 0.35 | C14H29NO | 69.0706, 69.9825, 79.2552, 88.0763, 185.491 |
| M100 | 26.38 | Cabraleahydroxylactone | [M-H]^-^ | 431.3165 | 431.3156 | -0.31 | C27H44O4 | 69.6054, 78.3045, 67.2807, 97.0814, 251.9095 |
| M101 | 26.76 | Dihydrodeoxy-8-epiaustdiol | [M+ACN+Na]^+^ | 286.1048 | 286.105 | -0.91 | C12H14O4 | 69.0705, 69.6765, 89.0796, 199.1438, 58.0659 |
| P38 | 26.79 | Stearic acid amide | [M+H]^+^ | 284.2947 | 284.295 | -0.34 | C18H37NO | 69.0705, 69.6343, 79.0548, 197.7162, 199.2953 |
| M102 | 26.94 | Echitamine | [M+NH4]^+^ | 403.2454 | 403.477 | -2.97 | C22H29N2O4 | 371.2194, 161.1581, 55.7465, 65.8712, 251.6302 |
| M103 | 27.08 | Lycojaponicuminol C | [M+H-H2O]^+^ | 461.3626 | 461.3625 | 0.24 | C29H48O4 | 69.0704, 79.055, 198.9455, 199.0265, 199.1478 |
| M104 | 27.15 | Prostaglandin F2alpha | [M+H-2H2O]^+^ | 319.2269 | 319.2268 | 0.27 | C20H34O5 | 69.0705, 79.0547, 89.0602, 199.1136, 58.8659 |
| M105 | 27.33 | Incensole | [M+NH4]^+^ | 324.2898 | 324.2897 | 0.2 | C20H34O2 | 69.0706, 79.0549, 195.3278, 199.1906, 88.2649 |
| M106 | 27.37 | 15-Deoxoeucosterol | [M+H]^+^ | 459.3472 | 459.3469 | 0.59 | C29H46O4 | 69.0707, 69.7812, 79.0549, 297.2217, 183.1178 |
| P39 | 27.48 | Tanshinone IIA | [M+Na]^+^ | 295.1326 | 295.133 | -0.97 | C19H18O3 | 199.6506, 199.6845, 161.6752, 65.9932, 53.3529 |
| M107 | 27.77 | Anacardic Acid | [M+H-H2O]^+^ | 331.2633 | 331.2632 | 0.41 | C22H36O3 | 69.0706, 79.0548, 295.2407, 183.1174, 187.1479 |
| M108 | 27.84 | Atractylochromene | [M+H]^+^ | 291.1956 | 291.1955 | 0.6 | C17H22O2 | 59.1771, 69.9469, 79.9037, 291.1955, 188.0837 |
| P40 | 28.68 | Androstane-3,6,17-triol | [M+H]^+^ | 309.2426 | 309.242 | 0.63 | C19H32O3 | 69.0707, 69.8209, 79.0549, 99.0808, 195.1373 |
| P41 | 28.71 | Lecithin | [M+K]^+^ | 796.5223 | 796.5253 | -3.93 | C42H80NO8P | 69.0706, 79.0547, 699.9788, 796.5259, 88.519 |
| P42 | 28.83 | Isodihydroauroglaucin | [M+H-H2O]^+^ | 283.1683 | 283.169 | -3.27 | C19H24O3 | 69.0706, 190.0769, 192.0926, 193.1013, 194.1087 |
| P43 | 29.18 | Germacrone | [M+H]^+^ | 219.1746 | 219.335 | 1.09 | C15H22O | 69.0341, 69.0706, 79.0549, 191.1798, 57.0343 |
| P44 | 29.25 | 9-cis-Retinal | [M+H]^+^ | 317.2477 | 318.255 | 0.66 | C20H28O | 69.0708, 69.677, 197.1329, 183.1171, 185.1331 |
| P45 | 29.46 | D-erythro-sphingosine | [M+H]^+^ | 300.2899 | 300.2897 | 0.66 | C18H37NO2 | 59.5074, 69.0707, 69.7673, 69.8204, 192.1509 |
| P46 | 29.92 | 2,6,6-Trimethyl-2,4-cycloheptadien-1-one | [M+H-H2O]^+^ | 133.1013 | 133.101 | 0.82 | C10H14O | 69.6453, 79.0549, 78.0472, 78.5347, 88.0221 |
| M109 | 30.02 | Ginkgolic acid C13:0 | [M+H-2H2O]^+^ | 285.2214 | 285.2213 | 0.39 | C20H32O3 | 69.0705, 79.055, 197.1328, 199.149, 183.1169 |
| M110 | 30.09 | 13,16,19-Docosatrienoic acid | [M+NH4]^+^ | 352.3212 | 352.321 | 0.71 | C22H38O2 | 59.032, 69.0706, 79.0218, 79.055, 195.1747 |
| M111 | 30.28 | Sterebin E | [M+H-2H2O]^+^ | 303.2319 | 303.2319 | 0.17 | C20H34O4 | 69.0706, 79.0548, 193.1225, 197.1334, 183.1173 |
| M112 | 30.68 | 26-Nor-8-oxo-alpha-onocerin | [M+H-H2O]^+^ | 427.3573 | 427.3571 | 0.64 | C29H48O3 | 69.0711, 79.0548, 79.842, 189.1631, 57.0707 |
| P47 | 30.71 | Evocarpine | [M+H]^+^ | 340.2619 | 339.256 | -4.68 | C23H33NO | 59.6489, 59.6733, 69.8435, 199.6115, 186.0916 |
| M113 | 30.91 | Lithocholic acid | [M-H]^-^ | 375.2904 | 375.2894 | -0.24 | C24H40O3 | 199.1476, 374.993, 375.2914, 65.9362, 252.2305 |
| M114 | 31.55 | 17-methylandrost-4-ene-3,6,17-triol | [M+H-H2O]^+^ | 303.2319 | 303.2319 | 0.24 | C20H32O3 | 69.0706, 69.4127, 79.0548, 198.2841, 198.8241 |
| M115 | 31.6 | 5-Heptadecylresorcinol | [M+FA-H]^-^ | 393.3012 | 393.2999 | 0.45 | C23H40O2 | 199.347, 393.2528, 393.3014, 182.7428, 188.1986 |
| M116 | 31.76 | 1-Stearoyl-sn-glycero-3-phosphocholine | [M+Na]^+^ | 546.3531 | 546.353 | 0.09 | C26H54NO7P | 79.0671, 68.2345, 487.2796, 57.0707, 176.992 |
| P48 | 31.79 | Docosahexaenoic Acid | [M+H]^+^ | 329.2473 | 329.2475 | -0.65 | C22H32O2 | 69.0705, 79.0549, 193.1227, 199.1483, 293.2245 |
| M117 | 31.85 | 6alpha-Hydroxyandrost-4-ene-3,17-dione | [M-H]^-^ | 301.1807 | 301.1798 | -0.74 | C19H26O3 | 59.0125, 59.106, 69.0332, 69.816, 99.4027 |
| M118 | 32.22 | Minaxin C | [M+2Na-H]^+^ | 587.2068 | 587.2075 | -1.23 | C26H38O12 | 69.5541, 195.0807, 195.1187, 197.0604, 199.2279 |
| M119 | 32.29 | 6alpha-Hydroxynidorellol | [M+H-H2O]^+^ | 305.2475 | 305.2475 | -0.16 | C20H34O3 | 69.0707, 79.0548, 191.1796, 193.1218, 185.1322 |
| M120 | 32.54 | Orobanone | [M+CH3OH+H]^+^ | 249.1849 | 249.1849 | 0.04 | C15H20O | 59.05, 69.0704, 79.0548, 197.7997, 199.1483 |
| M121 | 32.54 | Pregn-5-ene-3,17-diol | [M+ACN+H]^+^ | 360.2894 | 360.2897 | -0.94 | C21H34O2 | 69.0704, 79.0549, 58.0659, 67.0549, 178.1592 |
| M122 | 32.54 | (+)-Junenol | [M+H-H2O]^+^ | 240.2319 | 240.2322 | -1.5 | C15H26O | 69.0706, 79.055, 198.1859, 198.222, 184.1693 |
| M123 | 32.54 | Geranylacetone | [M+NH4]^+^ | 212.2008 | 212.2009 | -0.55 | C13H22O | 69.0706, 79.0547, 99.0807, 195.1743, 57.0342 |
| M124 | 32.75 | Gossyplure | [M+H-H2O]^+^ | 263.2372 | 263.2369 | 0.78 | C18H32O2 | 69.0706, 69.6318, 79.0549, 99.0809, 189.1643 |
| P49 | 32.75 | 4-Trimethylammoniobutanoic acid | [M+K]^+^ | 184.073 | 184.073 | -3.04 | C7H15NO2 | 59.0858, 58.9564, 98.9848, 184.0737, 56.0503 |
| M125 | 32.86 | Eicosapentaenoic acid ethyl ester | [M+H]^+^ | 331.2634 | 331.2632 | 0.82 | C22H34O2 | 69.0706, 79.0549, 195.1381, 185.1327, 187.1485 |
| M126 | 32.87 | Apocynol A | [M-H]^-^ | 223.1334 | 223.1329 | -2.6 | C13H20O3 | 59.0124, 69.0333, 195.1383, 182.9865, 183.3721 |

P: original blood-entry components; M: metabolites
